# Supplementary material for: From gHBfix to NBfix: Reweighting-Driven Refinement of Hydrogen-Bond Interactions in RNA Force Fields
Source: J Chem Theory Comput. 2026 Aug 16;22(17):9154–66. doi: 10.1021/acs.jctc.6c00924 (PMC13564359; doi:10.1021/acs.jctc.6c00924)
Supplement: Supplementary file 1 [file ct6c00924_si_001.pdf]

Supporting Information to From gHBfix to NBfix: Reweighting-Driven Refinement of Hydrogen-Bond Interactions in RNA Force Fields

Vojtěch Mlýnský,[1] Petra Kührová,[1,2] Giovanni Bussi,[3] Michal Otyepka,[2,4] Jiří Šponer,[1,2] and Pavel Banáš [2,4]

[1] Institute of Biophysics of the Czech Academy of Sciences, Královopolská 135, 612 00 Brno, Czech Republic

[2] Regional Center of Advanced Technologies and Materials, The Czech Advanced Technology and Research Institute (CATRIN), Palacký University Olomouc, Šlechtitelů 27, 779 00 Olomouc, Czech Republic

[3] Scuola Internazionale Superiore di Studi Avanzati, SISSA, via Bonomea 265, 34136 Trieste, Italy

[4] IT4Innovations, VSB – Technical University of Ostrava, 17. listopadu 2172/15, 708 00 Ostrava-Poruba, Czech Republic

Table of Contents

SUPPORTING RESULTS .... - 2 -

SUPPORTING TABLES .... - 4 -

SUPPORTING FIGURES .... - 7 -

REFERENCES .... - 22 -

SUPPORTING RESULTS

ParmEd file implementing the NBfix19 modification for the OL3$_{CP}$ RNA Force Field.[1-6]

ParmEd File Usage: $AMBERHOME/bin/parmed topologyfile_OL3CP.parm7 -i parmed_NBfix19.in

parmed_NBfix19.in

```
#
# parmed input file to modify off-diagonal Lennard-Jones parameters in order to replace external gHBfix19 H-bond correction potential (Kuehrova & Mlynsky et al., JCTC 2019) with the NBfix19 modification
# for details, see Mlynsky V., Kuehrova P., Bussi G., Otyepka M., Sponer J., and Banas P., 2026
#
# IMPORTANT: this assumes that the topology was built with OL3 RNA force field (Zgarbova et al., JCTC 2011) further adjusted by the phosphate oxygen van der Waals correction (CP) by Steinbrecher et al., JCTC 2012
# other force fields may have different atom types and need different atom masks below
#
#
# NBfix modification of -NH...N- interactions according to gHBfix19 potential
# all A_N6(H61/H62), C_N4(H41/H42), G_N2(H21/H22), G_N1(H1), and U_N3(H3) hydrogens share same and unique atom type H
# A(N1), A(N3), A(N7), C(N3), G(N3), and G(N7) share same NB and NC atom types, but these are also share with, e.g., N, NA, N2 .., so we need to split it
addLJType @%NB,NC
changeLJPair @%NB,NC @%H 2.1 0.63
#
# NBfix modification of -OH...bO/nbO- interactions according to gHBfix19 potential
# all 2'-OH hydrogens (i.e., HO2', HO3', and HO5') share same and unique atom type HO
# all bridging (bO; O5' and O3') and nonbridging (nbO; O1P and O2P) phosphate oxygens share same and unique atom types OR and OP
changeLJPair @%HO @%OP 2.73 0.01
changeLJPair @%HO @%OR 3.03 0.01
#
#
# CHANGE the newtopologyfile to your new name to output a parm7 file with the NBfix19 modification
outparm newtopologyfile_NBfix19.parm7
```

Additional UUCG tetraloop simulations with newly derived reparameterization of the OL3 Force Field. During completing results for this work, machine-learning–assisted reparameterization of the OL3 Force Field (FF), which incorporates experimental data into fitting schemes, has been published by Pak and coworkers (denoted here as OL3-vdW7-PAK)[7]. The authors presented very encouraging results considering 10-mer r(cacUUCGgug) tetraloop (UUCG TL) derived from the 14-mer NMR structure (PDB ID 2KOC[8]), showing dominant population of the native state during multiple-walker on-the-fly probability (OPES)[9, 10] enhanced-sampling simulations.[7]

Here, we performed a set of four independent 10 µs-long standard MD simulations for both shorter r(gcUUCGgc) 8-mer and longer r(cacUUCGgug) 10-mer TLs with the OL3-vdW7-PAK FF. Starting structures were taken from our previous work[11] and excised from the NMR structure (snapshot #13)[8] for the shorter 8-mer and longer 10-mer TL, respectively. Simulations were performed in Gromacs2020[12] with FF libraries published by the original authors (directly downloaded from the Zenodo repository: https://zenodo.org/records/15622306).[7] Systems were solvated with the OPC3 water model.[13] KCl ions described by Li and Merz parameters[14] for the OPC3 water were added to neutralize the system and to establish excess-salt ion concentration of 0.15 M. Gromacs simulations were performed in a rhombic dodecahedral box and bonds involving hydrogens were constrained using the LINCS algorithm.[15] The cut-off distance for the direct space summation of the electrostatic interactions was 10 Å and the simulations were performed at 298 K using the stochastic velocity rescale thermostat.[16]

Surprisingly, our results from the standard MD simulations showed that the UUCG TL is very poorly described by the OL3-vdW7-PAK FF, in sharp contrast with the original paper.[7] We observed almost immediate and irreversible loss of the signature interactions with the loop within first few hundreds of ns in all four attempted MD simulations of the shorter 8-mer TL (Figure S21). Comparable behavior was observed during simulations of the longer 10-mer TL, where the stability of the native state was slightly prolonged towards ~2 µs (in 3 out of 4 MD simulations; Figure S22). The A-form stem containing two (8-mer) or three (10-mer) canonical base pairs remained stable in all simulations for the entire 10 µs-long timescale. The irreversible disruption of the native state was initiated by the key G nucleotide (G6 and G7 for 8-mer and 10-mer, respectively) of the loop with *syn* conformation of χ dihedral, which left the binding site and flipped into *anti* state. This is typical and well documented disruption pathway of contemporary RNA FFs.[17, 18]

SUPPORTING TABLES

Table S1. Clustering analysis of 10 µs-long standard MD simulations of five TNs.[a]

System	Cluster	OL3$_{CP}$–gHBfix19	OL3$_{CP}$–NBfix19
r(AAAA)	A-form	28.9	25.6
	Intercalated	-	1.0
	Bulge-out	15.5	2.2
	Flipped	24.3	34.9
	1-2/3-4 stack	1.3	-
	Loop	1.5	-
	1-3/2-4 stack	-	9.7
r(CAAU)	A-form	29.7	47.6
	Intercalated	24.5	-
	Flipped	-	3.9
	1-2/3-4 stack	-	1.1
	Bulge-out	23.2	31.0
r(CCCC)	A-form	81.5	65.8
	Intercalated	-	24.5
	Bulge-out	6.9	2.0
	1-3/2-4 stack	-	3.4
r(GACC)	A-form	88.0	80.5
	Loop	4.4	-
	Bulge-out	-	9.5
r(UUUU)	A-major	4.4	6.8
	Intercalated	4.3	3.4
	1-2/3-4 stack	2.0	7.4
	1-3/2-4 stack	34.0	29.8
	Bulge-out	23.4	19.4
	Loop	9.7	2.6
	Flipped	1.4	-

[a] See Methods in the main text for details about clustering algorithm and Ref.[19] for details about structural states.

Table S2. Complementary clustering analysis of 10 µs-long standard MD simulations of five TNs.[a]

System	Cluster	OL3$_{CP}$–gHBfix19	OL3$_{CP}$–NBfix19
r(AAAA)	A-major	12.5	15.2
	A-minor	3.8	4.2
	Intercalated	0.1	0.0
r(CAAU)	A-major	17.5	30.5
	A-minor	6.1	10.1
	Intercalated	22.3	0.7
r(CCCC)	A-major	58.0	54.9
	A-minor	8.2	5.0
	Intercalated	1.8	24.3
r(GACC)	A-major	67.4	47.3
	A-minor	8.1	6.1
	Intercalated	0.2	3.2
	Stack	0.0	6.1
	Loop	4.3	0.0
r(UUUU)	A-major	4.2	6.5
	A-minor	13.1	9.5
	Intercalated	3.4	2.4

[a] Targeted εRMSD-based state assignment was applied for predefined cluster types, where representative PDB structures of key conformations were used as references (see Methods in the main text for details and Ref.[19] for details about structural states).

Table S3. Clustering analysis of REST2 simulations of five TNs.[a]

System	Cluster	$OL3_{CP}$–gHBfix19	$OL3_{CP}$–NBfix19
r(AAAA)	A-form	28.8	28.7
	Intercalated	11.3	8.9
	Bulge-out	8.8	3.7
	Flipped	20.5	39.2
	1-2/3-4 stack	4.6	-
	1-3/2-4 stack	3.9	2.2
r(CCCC)	A-form	55.3	71.1
	Intercalated	40.4	23.3
	Bulge-out	-	1.1
r(UUUU)	A-form	-	8.7
	1-3/2-4 stack	31.4	38.2
	Bulge-out	12.1	18.3
	Flipped	13.3	12.7
	Unstructured	15.5	3.3
r(CAAU)	A-form	33.4	49.9
	Intercalated	51.4	31.4
	Bulge-out	4.4	5.8
r(GACC)	A-form	73.5	86.6
	Intercalated	1.9	-
	Loop	7.0	2.2
	Bulge-out	5.5	1.7

[a] All simulations used 8 replicas and were run for 10 μs (the last 7 μs were used for data analysis). See Methods in the main text for details about clustering algorithm and Ref.[19] for details about structural states.

Table S4. Averaged values of global helical parameters (and their standard deviations) from 10 μs-long standard MD simulations of the r(GCACCGUUGG)$_2$ decamer excised from the 1QC0[20] structure and of the r(UUAUAUAUAUAUAA)$_2$ tetradecamer 1RNA.[21] [a]

System/Setup	Helical rise [Å]	Helical incl. [°]	Tip [°]	Helical twist [°]	Major width [Å]	Minor width [Å]	X-disp. [Å]	Y-disp [Å]
1RNA(Exp.)	2.6	18.8	-0.4	32.7	18.3	13.3	-4.1	-0.1
$OL3_{CP}$–gHBfix19	2.5 ± 0.1	18.2 ± 0.8	0.0 ± 0.1	33.0 ± 1.1	19.2 ± 0.8	13.3 ± 0.4	-4.2 ± 0.1	0.0 ± 0.0
$OL3_{CP}$–NBfix19	2.5 ± 0.0	18.2 ± 0.5	0.0 ± 0.1	33.1 ± 0.2	19.2 ± 0.0	13.3 ± 0.0	-4.1 ± 0.1	0.0 ± 0.0
1QC0 (Exp.)	2.5	16.9	1.0	32.1	18.5	13.1	-4.6	0.0
$OL3_{CP}$–gHBfix19	2.7 ± 0.0	15.6 ± 0.6	-0.5 ± 0.1	31.9 ± 0.2	19.2 ± 0.0	13.4 ± 0.0	-4.7 ± 0.0	0.0 ± 0.0
$OL3_{CP}$–NBfix19	2.7 ± 0.0	15.4 ± 0.6	-0.4 ± 0.1	31.8 ± 0.2	19.2 ± 0.0	13.4 ± 0.0	-4.7 ± 0.1	0.0 ± 0.3

[a] Simulation were run with the reference $OL3_{CP}$–gHBfix19 and $OL3_{CP}$–NBfix19 FFs (see Methods in the main text). The first two base pairs from each termini were excluded from the calculations to prevent end effects. Standard deviations were calculated using block average over 1000 snapshots (saved every 10 ps).

Table S5. Averaged values of base pair and local base pair step helical parameters (and their standard deviations) from 10 μs standard MD simulations of the r(GCACCGUUGG)$_2$ decamer excised from the 1QC0[20] structure and of the r(UUAUAUAUAUAUAA)$_2$ tetradecamer 1RNA.[21] [a]

System/Setup	Shear [Å]	Stretch [Å]	Stagger [Å]	Buckle [°]	Propeller [°]	Opening [°]	Tilt [°]	Shift [Å]	Slide [°]	Rise [Å]	Roll [°]	Twist [°]	Fraying (top, bottom) [b] [%]	
1RNA(Exp.)	-0.1	-0.2	0.0	1.3	-18.8	-0.4	0.1	0.1	-1.3	3.3	10.0	30.5	-	-
$OL3_{CP}$–gHBfix19	0.0 ± 0.0	0.0 ± 0.0	0.1 ± 0.0	0.0 ± 0.2	-17.4 ± 0.6	0.9 ± 0.2	0.0 ± 0.1	0.0 ± 0.0	-1.5 ± 0.1	3.2 ± 0.1	10.5 ± 0.4	30.2 ± 1.0	3.9	5.4
$OL3_{CP}$–NBfix19	0.0 ± 0.0	0.0 ± 0.0	0.1 ± 0.0	0.0 ± 0.2	-17.4 ± 0.2	0.9 ± 0.2	0.0 ± 0.1	0.0 ± 0.0	-1.5 ± 0.0	3.2 ± 0.0	10.5 ± 0.3	30.2 ± 0.1	4.9	4.6
1QC0 (Exp.)	0.1	-0.3	0.1	1.2	-12.5	1.4	-0.6	0.0	-1.7	3.2	9.0	30.4	-	-
$OL3_{CP}$–gHBfix19	0.0 ± 0.0	0.0 ± 0.0	-0.1 ± 0.0	1.0 ± 0.2	-12.4 ± 0.5	-0.1 ± 0.1	0.2 ± 0.1	0.0 ± 0.0	-1.8 ± 0.0	3.3 ± 0.0	8.6 ± 0.4	29.9 ± 0.2	0.1	0.3
$OL3_{CP}$–NBfix19	0.0 ± 0.0	0.0 ± 0.0	-0.1 ± 0.0	1.0 ± 0.2	-12.3 ± 0.5	0.0 ± 0.1	0.2 ± 0.1	0.0 ± 0.0	-1.8 ± 0.0	3.3 ± 0.0	8.5 ± 0.3	29.8 ± 0.3	0.1	0.2

[a] Simulation were run with the reference $OL3_{CP}$–gHBfix19 and $OL3_{CP}$–NBfix19 FFs (see Methods in the main text). The first two base pairs from each termini were excluded from the calculations to prevent end effects. Standard deviations were calculated using block average over 1000 snapshots (saved every 10 ps).
[b] Top and bottom base pairs are G_1C_{20} and $G_{10}C_{11}$ for the 1QC0 duplex and U_1A_{28} and $A_{14}U_{15}$ for the 1RNA duplex, respectively.

SUPPORTING FIGURES

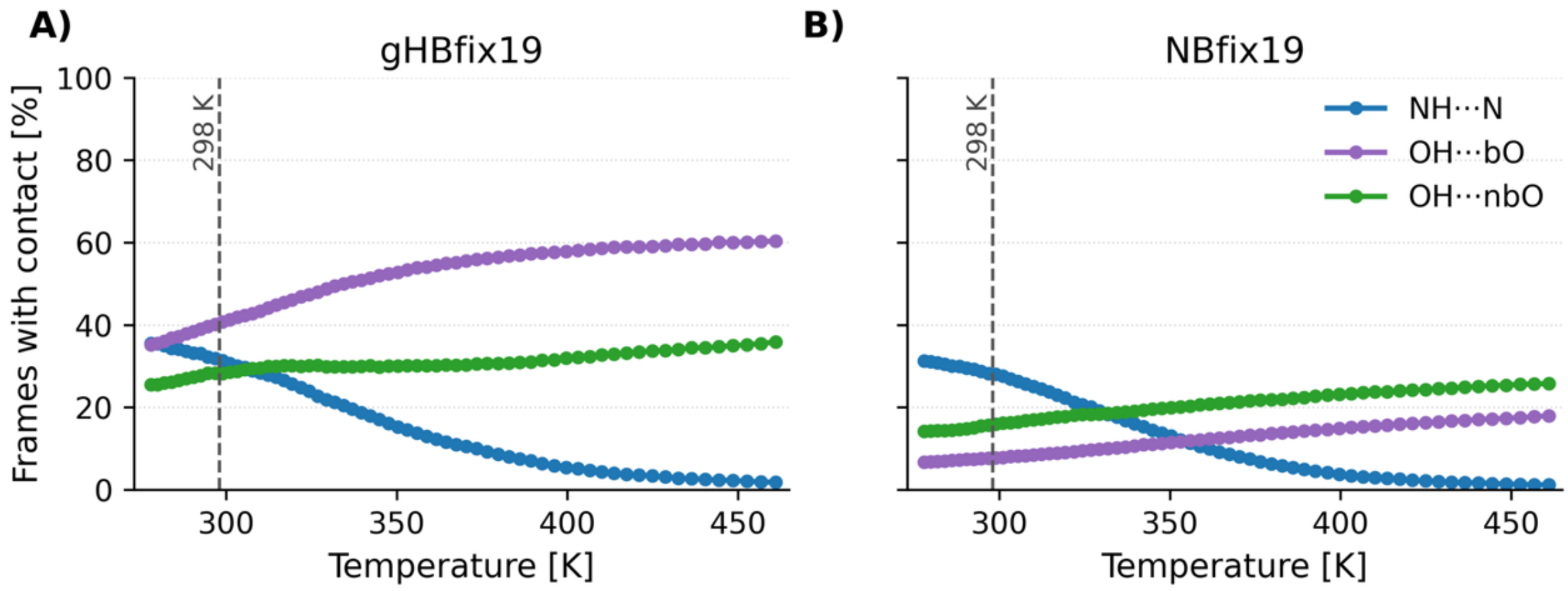

Figure S1. Temperature dependence of contact populations in the GAGA TL from T-REMD simulations using the OL3$_{CP}$–gHBfix19 (gHBfix19) and OL3$_{CP}$–NBfix19 (NBfix19) FFs. For the gHBfix19 simulation, only the last 5 µs of each replica trajectory were included in the analysis. Contact populations are reported as the percentage of frames containing at least one contact belonging to the indicated chemical H-bond class, i.e., –NH···N–, –OH···bO–, and –OH···nbO–, identified using the corresponding donor-hydrogen–acceptor distances. The dashed vertical line marks 298 K. Note that, unlike gHBfix19, NBfix19 applies the nonbonded correction also to the vicinal 2'-OH···O3' interaction within the same ribose, as discussed in the main text.

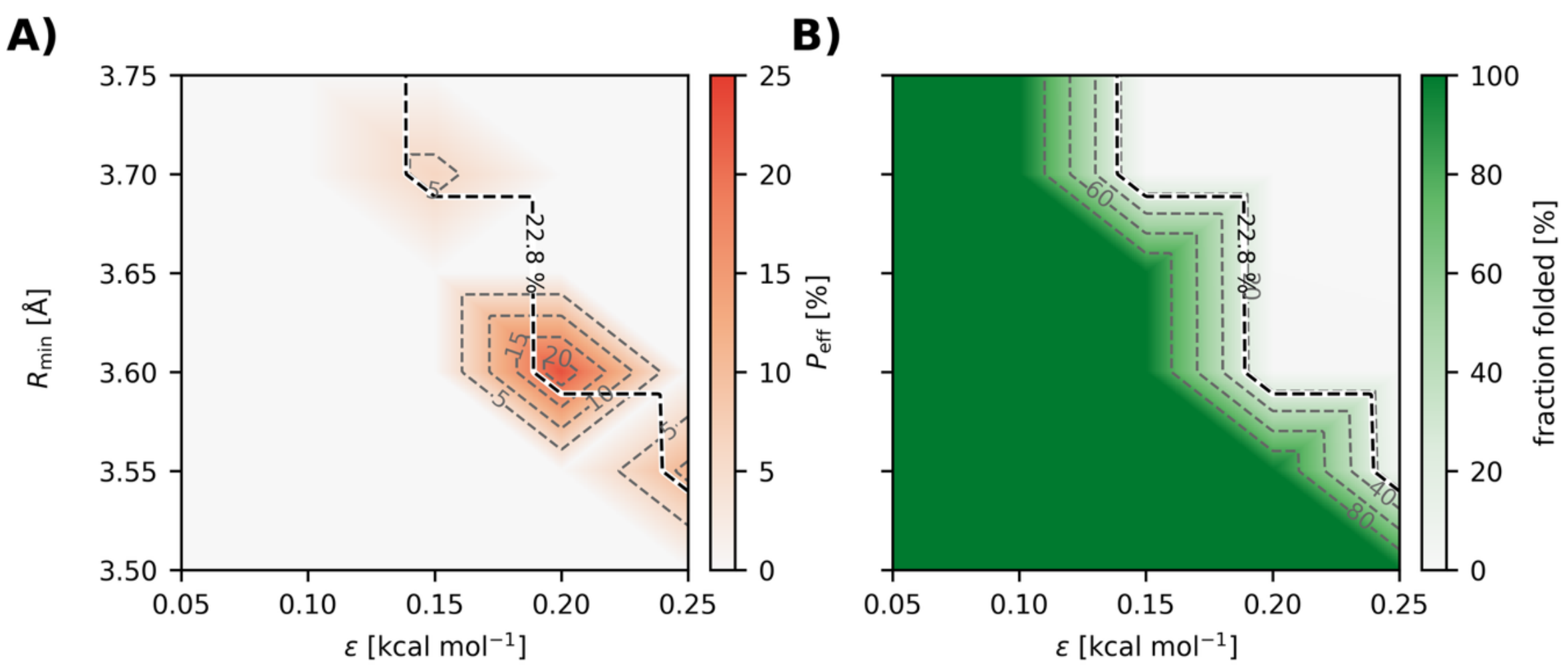

Figure S2: Reweighting-based optimization of NBfix parameters representing the gHBfix19 –NH···N– correction through the heavy-atom donor-N/acceptor-N Lennard–Jones (LJ) pair. (A) Normalized effective sample size (P_{eff}) of the reweighted ensemble as a function of the LJ radius (R) and well depth (ε). (B) Reweighted folded-state population over the same parameter range, with the dashed contour indicating the target folded-state population obtained from the converged OL3$_{CP}$–gHBfix19 T-REMD simulation.

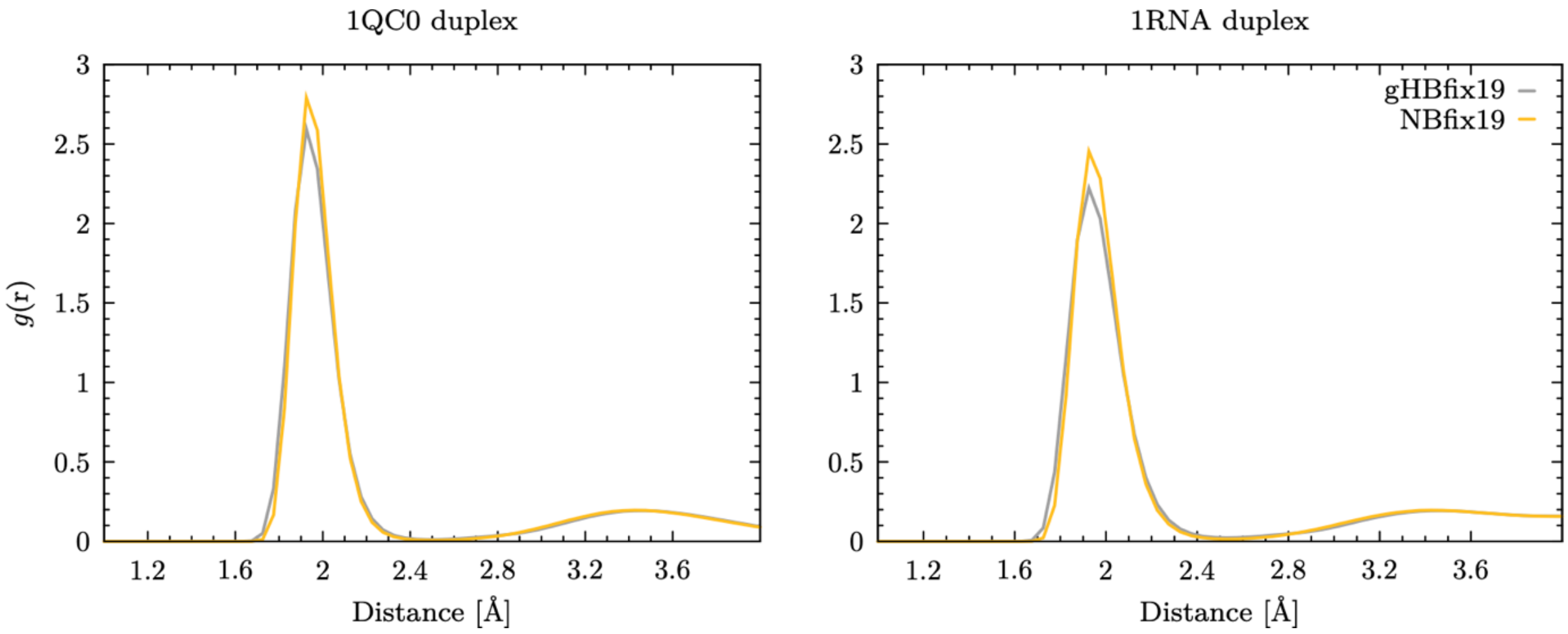

Figure S3: Radial distribution functions *g*(r) for –NH···N– interactions, evaluated using the corresponding –H···N– distances, in the canonical RNA duplexes 1QC0 and 1RNA for the reference $OL3_{CP}$–gHBfix19 and optimized $OL3_{CP}$–NBfix19 FFs.

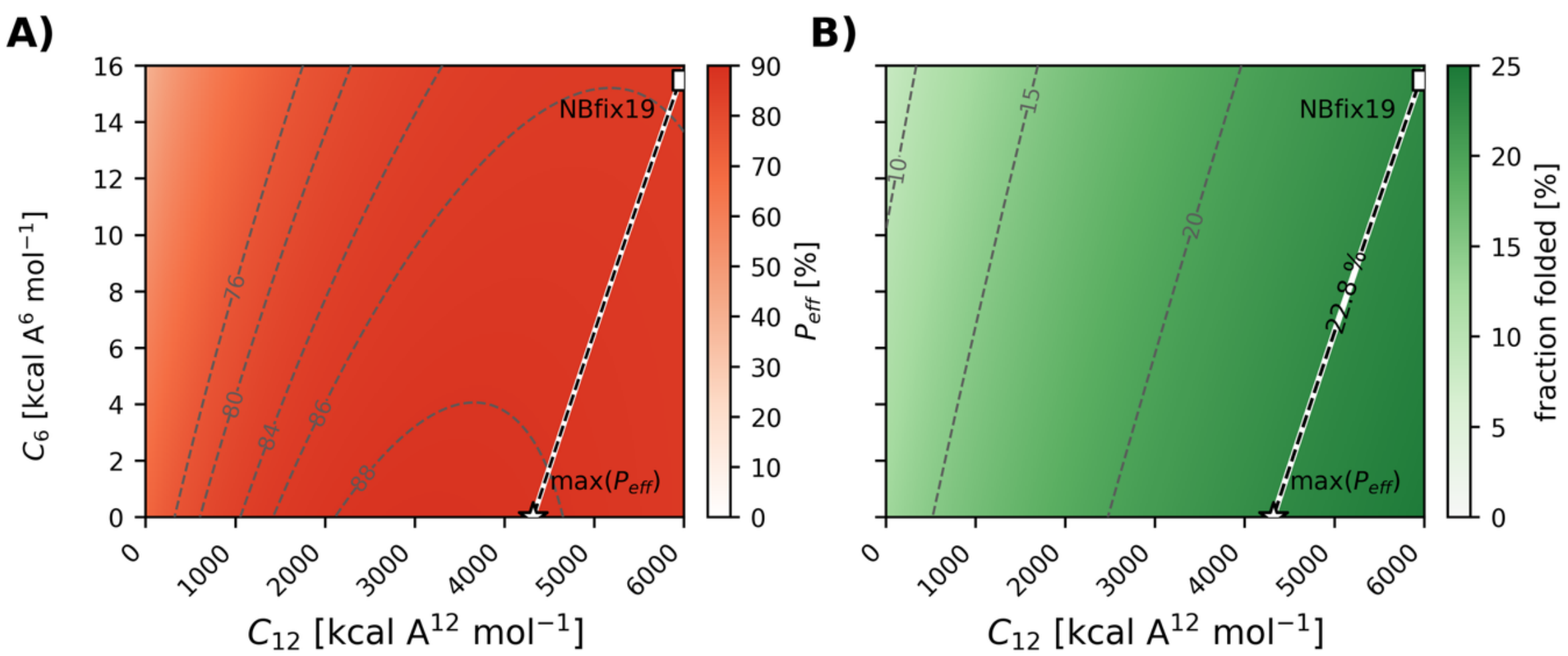

Figure S4. Reweighting-based optimization of NBfix C_6/C_{12} parameters representing the gHBfix19 –OH···bO– correction. (A) Normalized effective sample size (P_{eff}) of the reweighted ensembles as a function of C_6 and C_{12} parameters. (B) Corresponding reweighted folded-state fractions. The dashed contour indicates the target folded-state fraction obtained from the converged $OL3_{CP}$–gHBfix19 T-REMD simulation. The star marks the point on the target contour with the maximum P_{eff}, and the square denotes the selected NBfix19 parameter values.

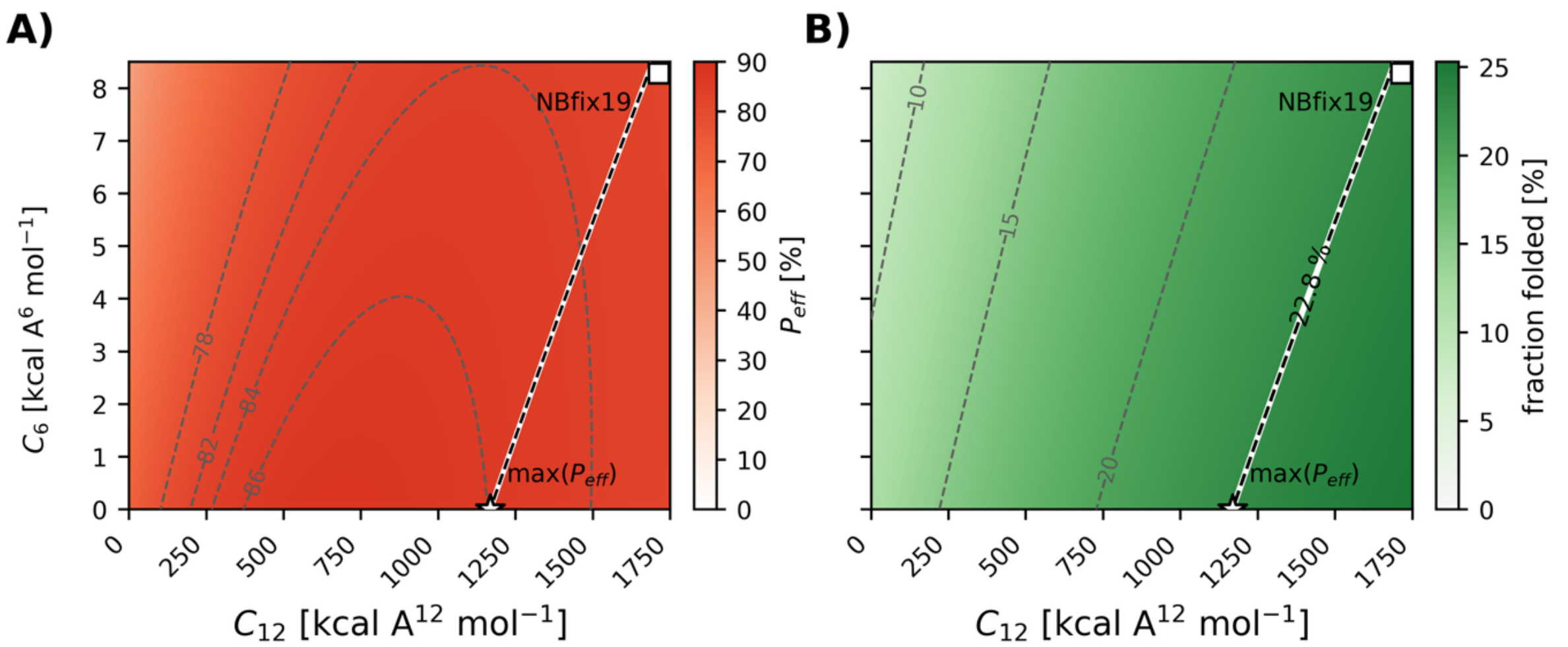

Figure S5. Reweighting-based optimization of NBfix C_6/C_{12} parameters representing the gHBfix19 –OH···nbO– correction. The definitions of P_{eff}, the folded-state fraction, and the symbols are identical to those described for Figure S4.

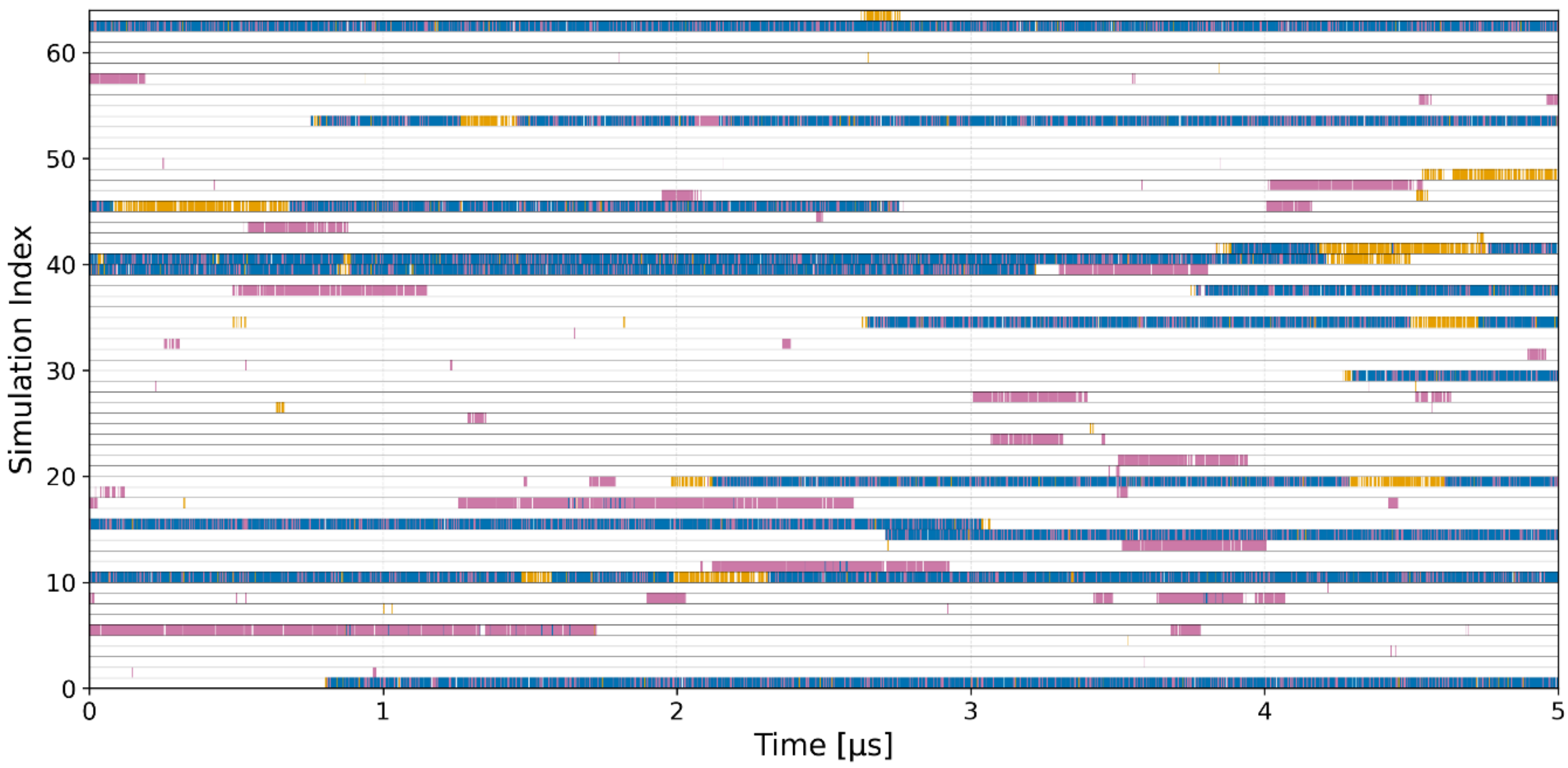

Figure S6. Conformational sampling of T-REMD folding simulation of the GAGA TL using the $\mathrm{OL3_{CP}}$–gHBfix19–$\mathrm{NBfix_{NH...N}}$ FF variant. The panel shows time evolution of major conformers, i.e., (i) correctly folded A-form stem and loop (native states with all signature interactions formed, blue), (ii) folded A-form stem (loop not in native conformation, purple), and (iii) correctly folded loop (stem not in A-form, orange), in all 64 continuous (demultiplexed) trajectories.

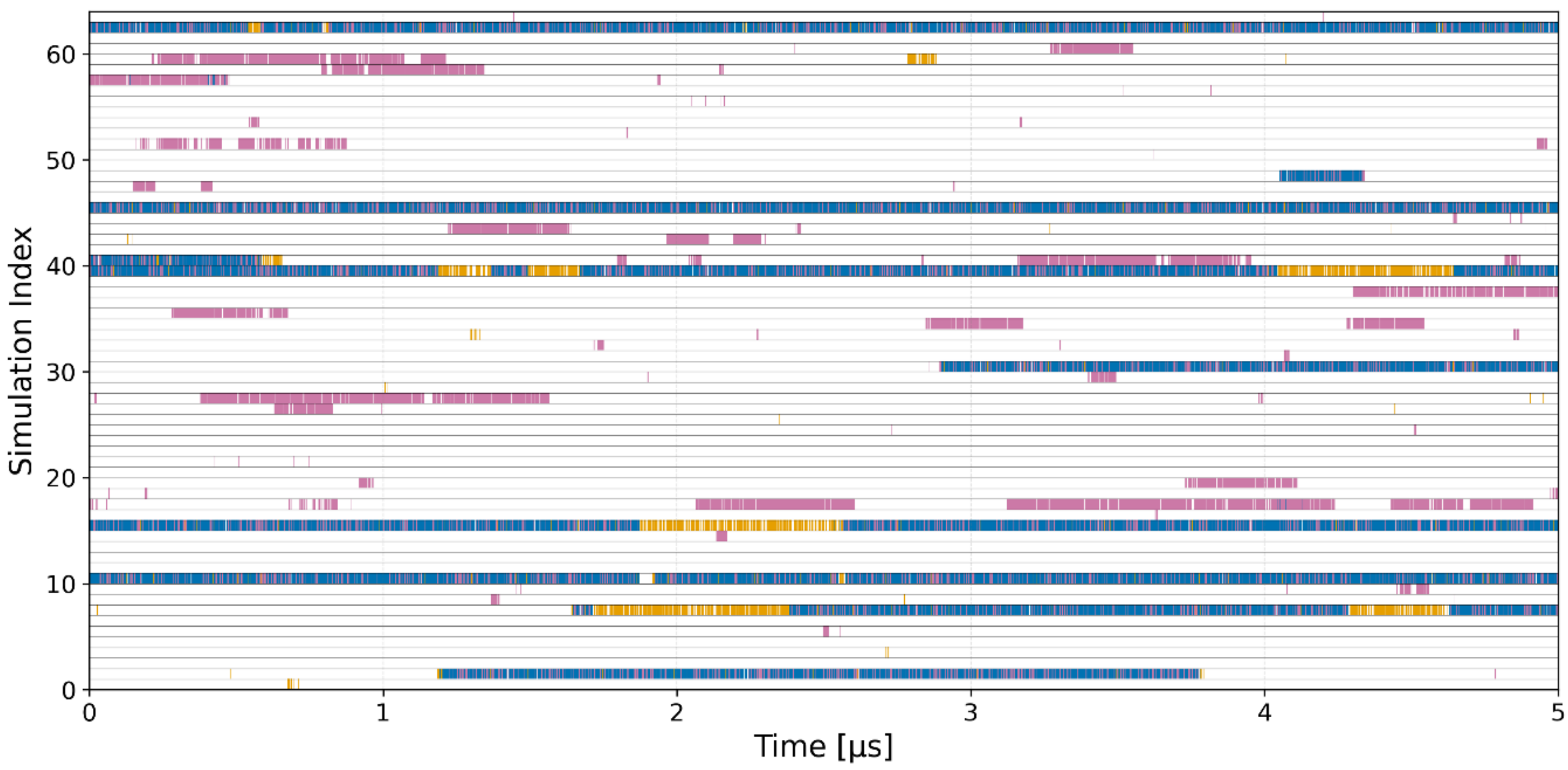

Figure S7. Conformational sampling of T-REMD folding simulation of the GAGA TL using the $OL3_{CP}$–gHBfix19–$NBfix_{OH...nbO}$ FF variant. See Figure S6 for more details.

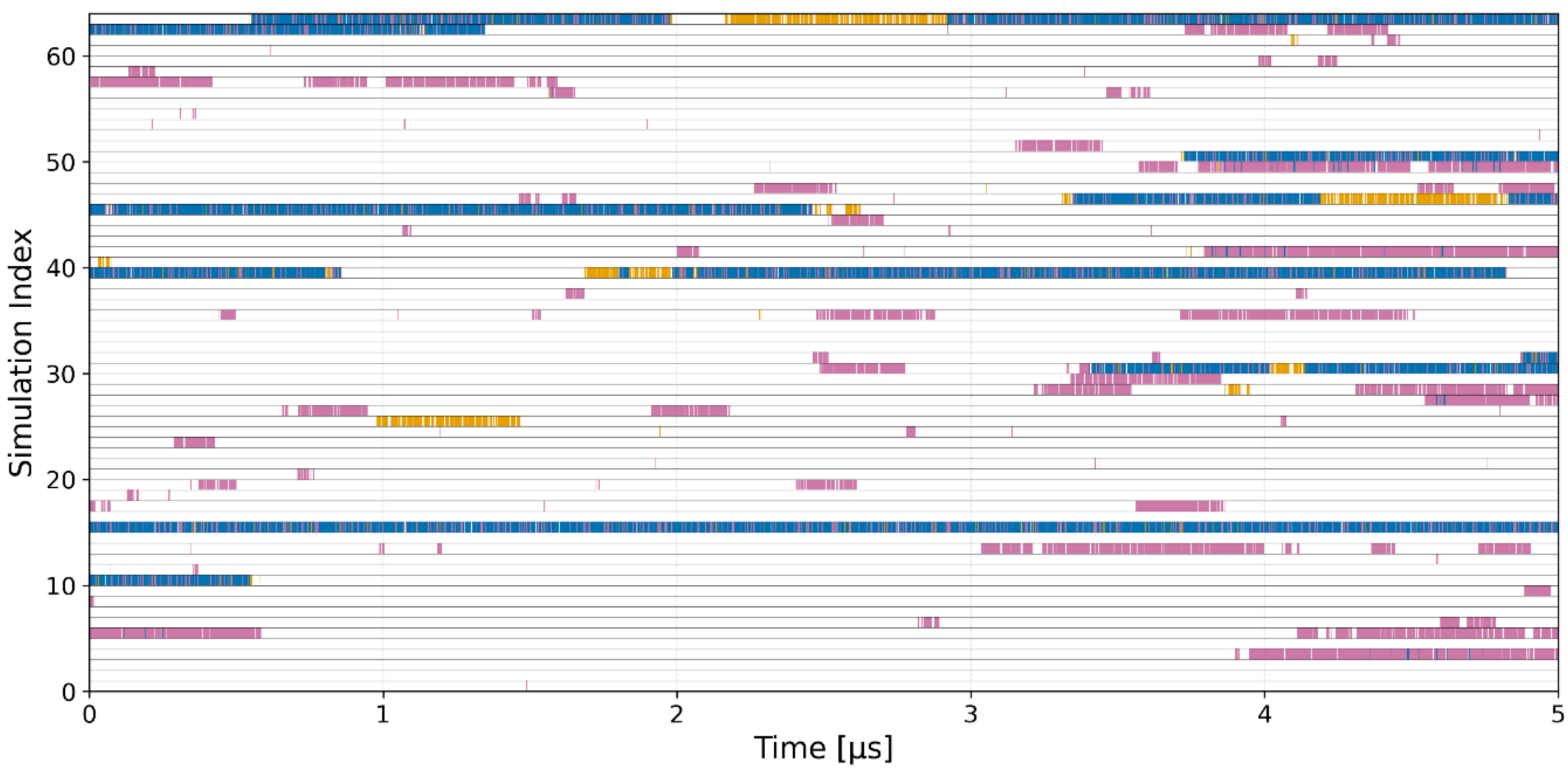

Figure S8. Conformational sampling of T-REMD folding simulation of the GAGA TL using the $OL3_{CP}$–gHBfix19–$NBfix_{OH...bO}$ FF variant. See Figure S6 for more details.

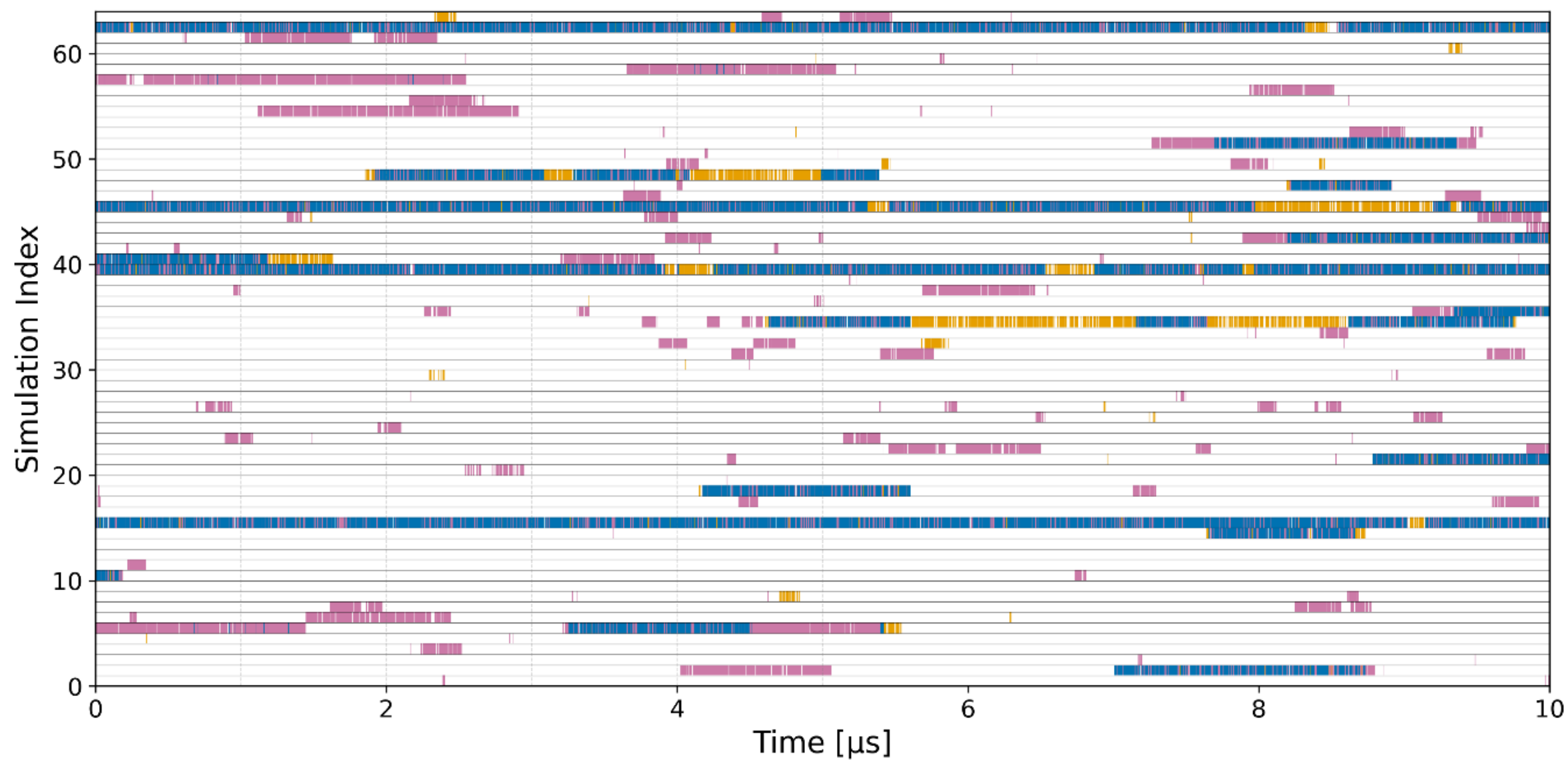

Figure S9. Conformational sampling of T-REMD folding simulation of the GAGA TL using the OL3CP–NBfix19 FF. See Figure S6 for more details.

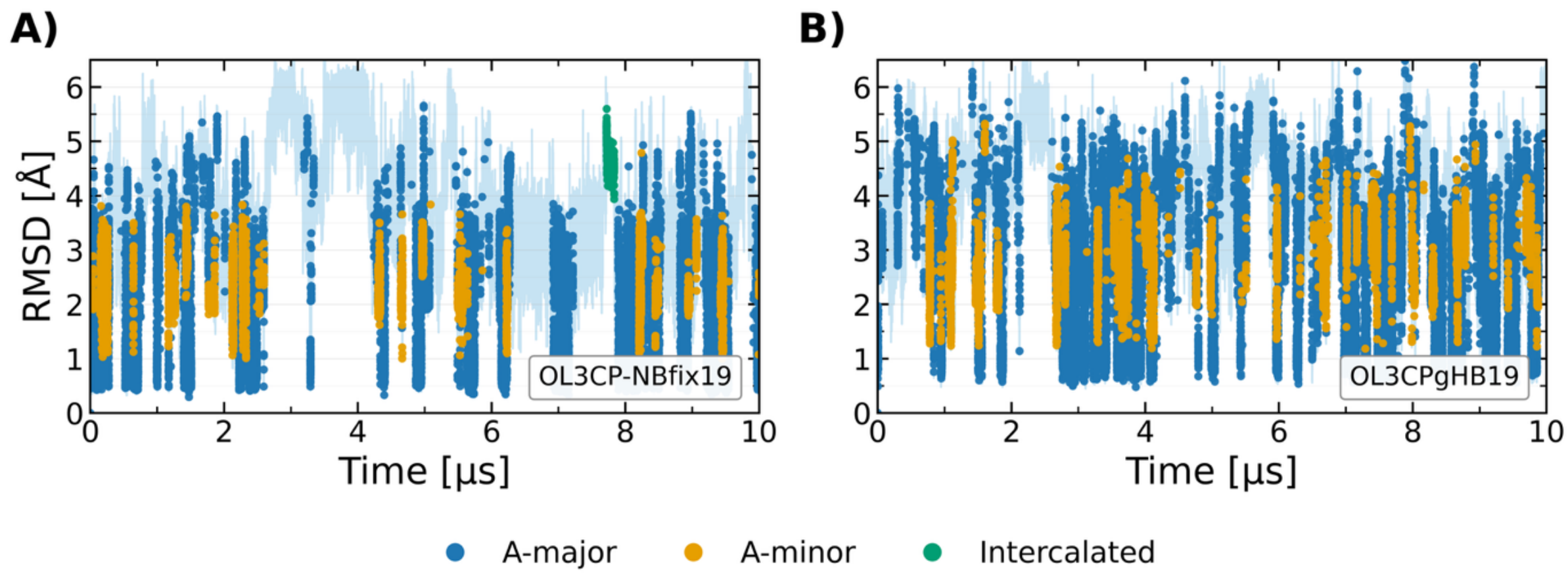

Figure S10. Time evolution of the RMSD with respect to the A-form conformation in two 10 µs-long standard MD simulations of the r(AAAA) TN using (A) $OL3_{CP}$–NBfix19 and (B) $OL3_{CP}$–gHBfix19 FFs. Light-blue traces show the RMSD time series. Colored markers denote frames assigned to reference conformational clusters based on the minimum εRMSD[22] to the cluster templates (assignment threshold εRMSD = 0.7): A-major (blue), A-minor (gold), and Intercalated (green). Frames that do not satisfy the assignment threshold are not classified and are therefore not shown.

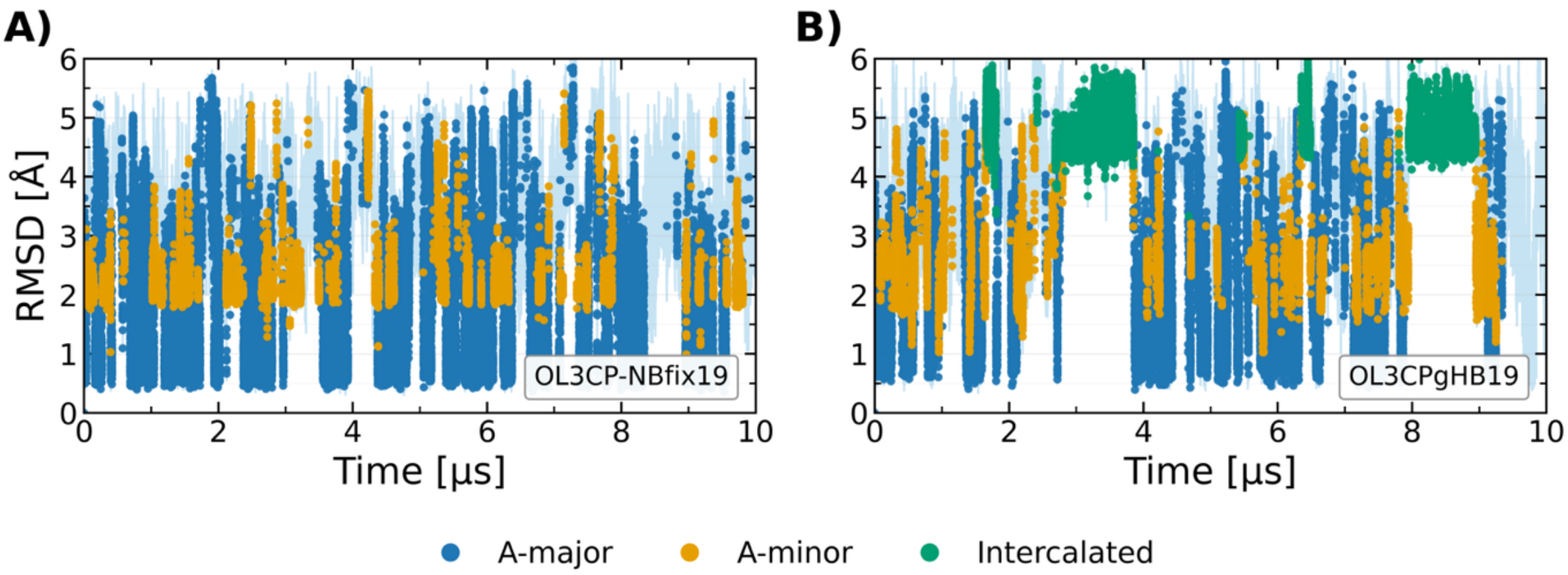

Figure S11. Time evolution of the RMSD in two 10 µs-long standard MD simulations of the r(CAAU) TN using (A) $OL3_{CP}$–NBfix19 and (B) $OL3_{CP}$–gHBfix19 FFs. See Figure S10 for more details.

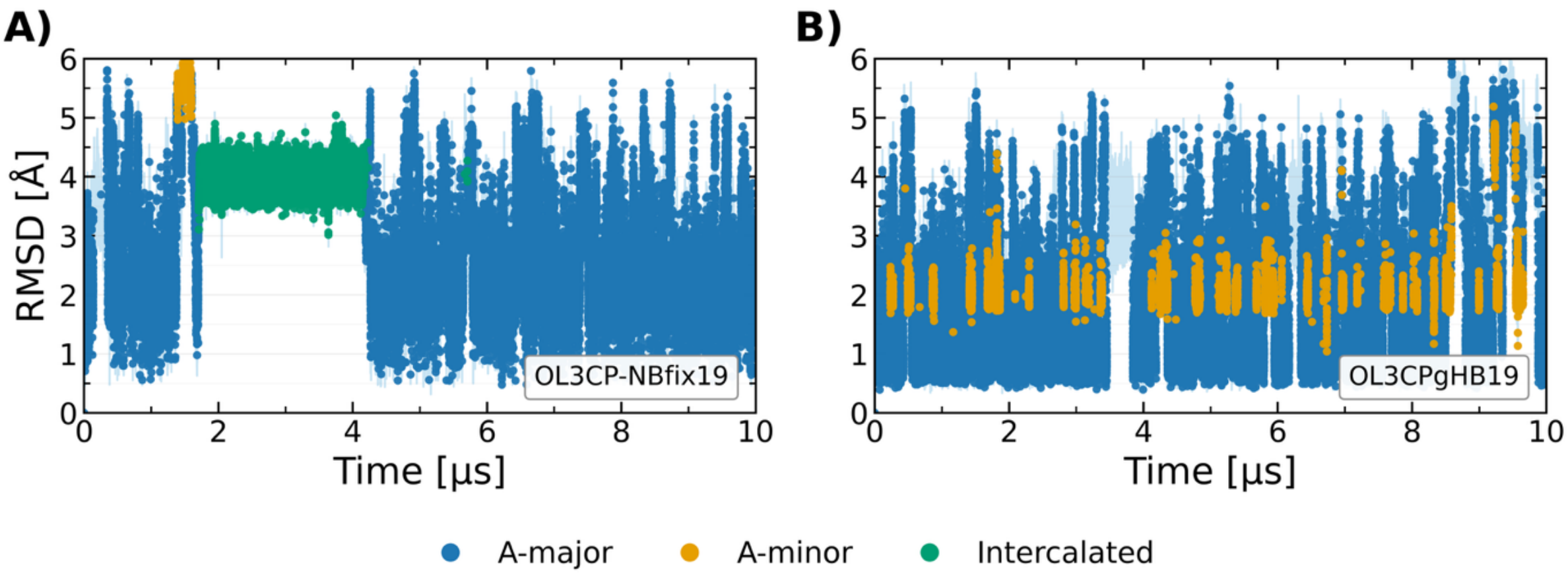

Figure S12. Time evolution of the RMSD in two 10 µs-long standard MD simulations of the r(CCCC) TN using (A) $OL3_{CP}$–NBfix19 and (B) $OL3_{CP}$–gHBfix19 FFs. See Figure S10 for more details.

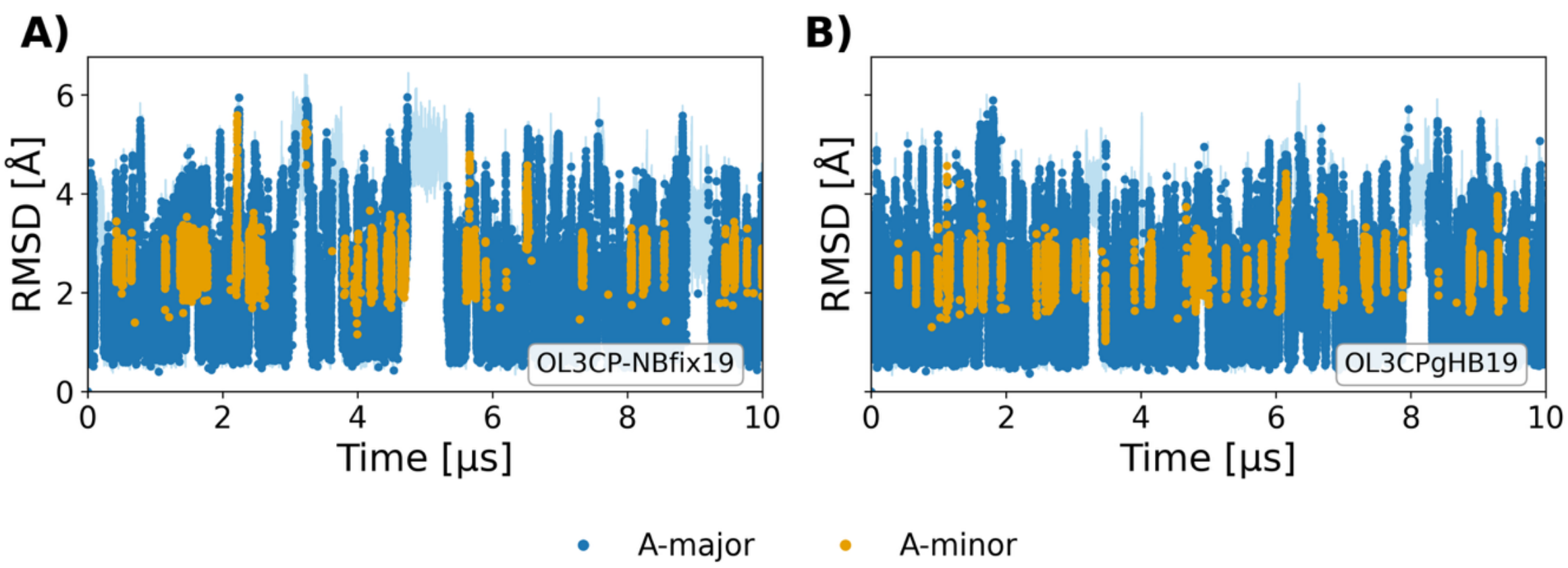

Figure S13. Time evolution of the RMSD in two 10 µs-long standard MD simulations of the r(GACC) TN using (A) $OL3_{CP}$–NBfix19 and (B) $OL3_{CP}$–gHBfix19 FFs. See Figure S10 for more details.

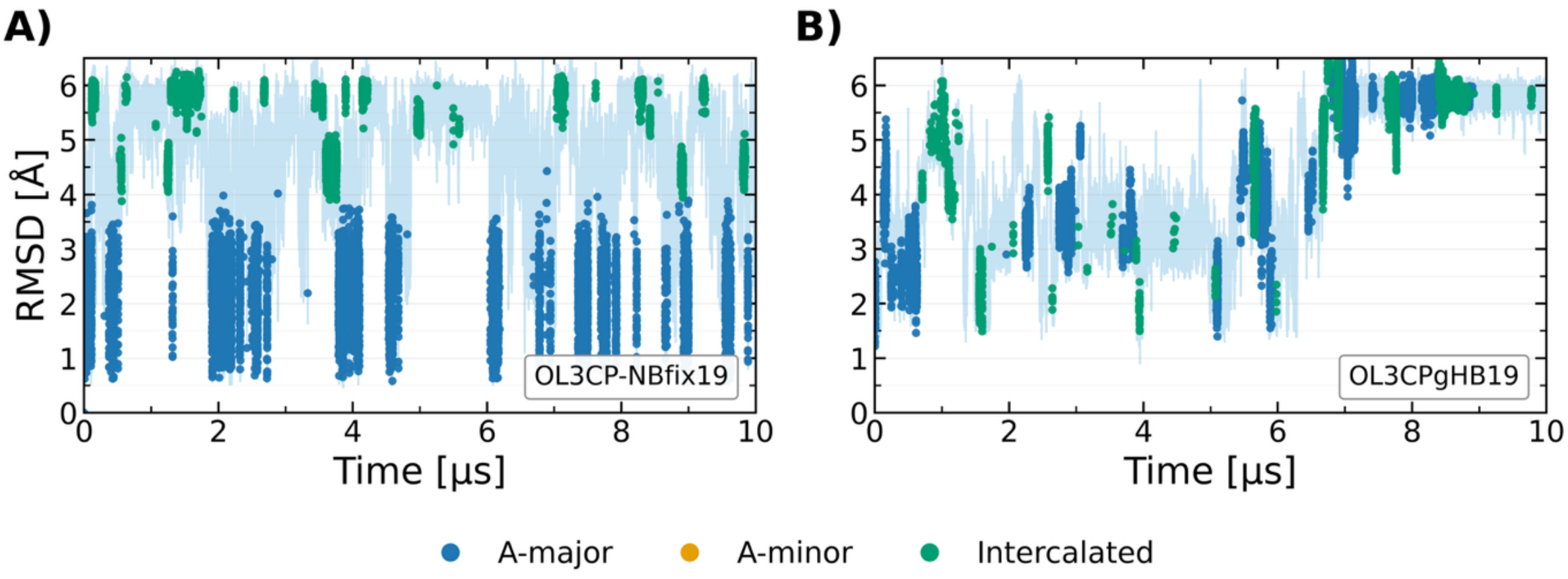

Figure S14. Time evolution of the RMSD in two 10 µs-long standard MD simulations of the r(UUUU) TN using (A) $OL3_{CP}$–NBfix19 and (B) $OL3_{CP}$–gHBfix19 FFs. See Figure S10 for more details.

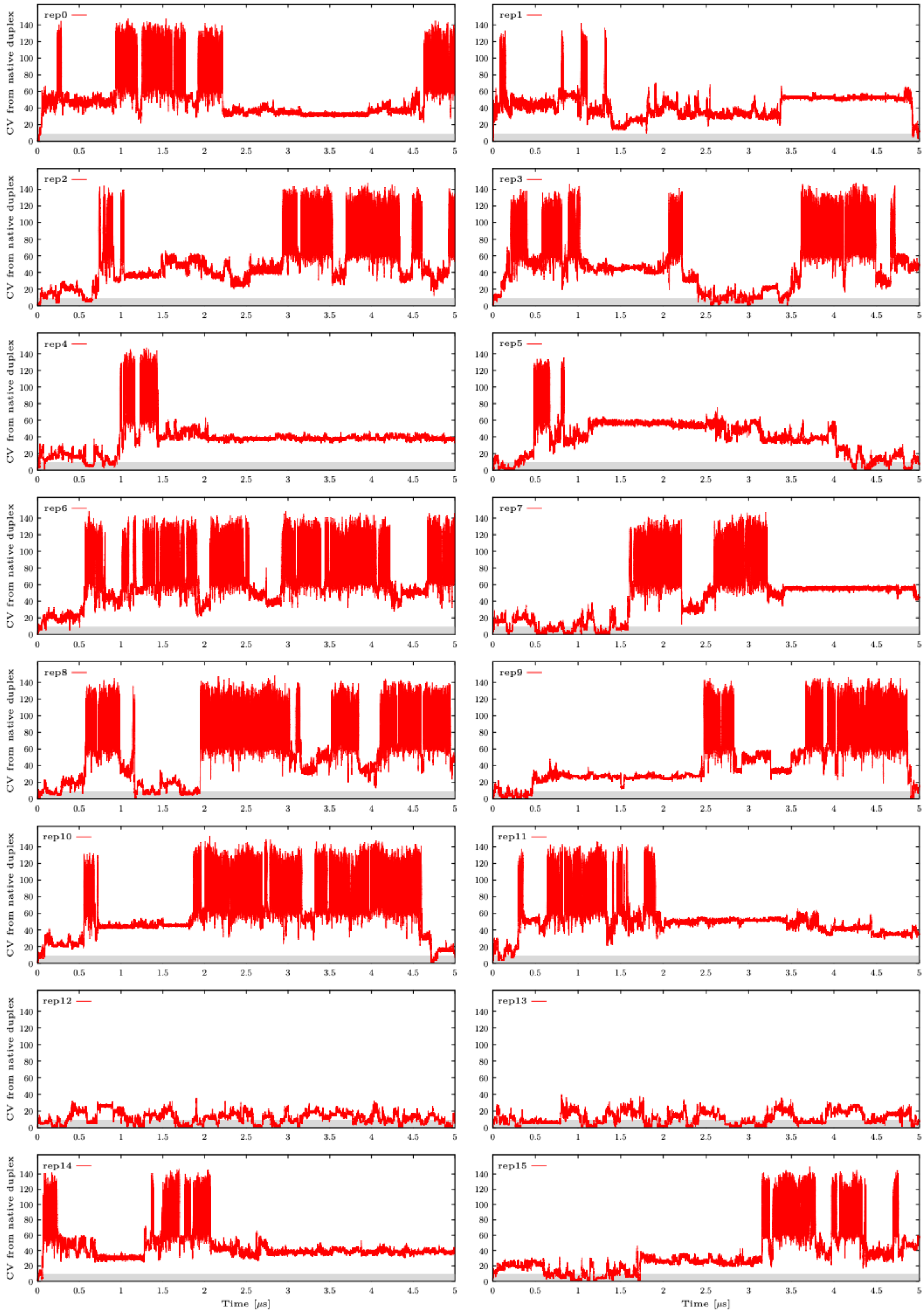

Figure continuing on the next page

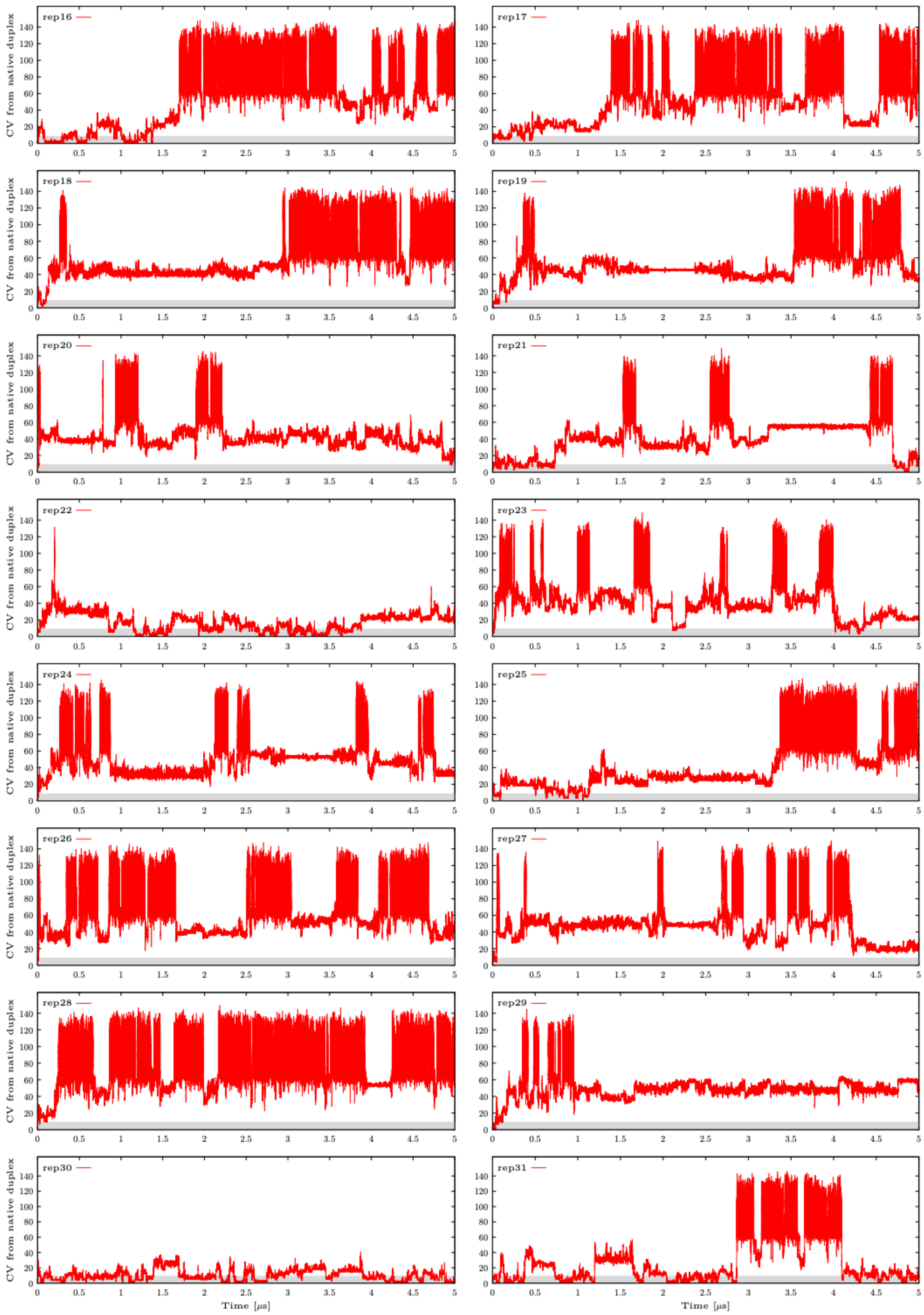

Figure S15. Calculated CV (a combination of RMSD and εRMSD; see Methods in the main text) relative to the native state for each of the 32 continuous (demultiplexed) replicas from the 5 μs PT-OPES simulation of the short r(GGCC)$_2$ duplex with the OL3$_{CP}$–gHBfix19 FF. CV values were evaluated every 50 ps. The shaded region highlights conformations belonging to the folded (native-like) ensemble (CV < 8.9).

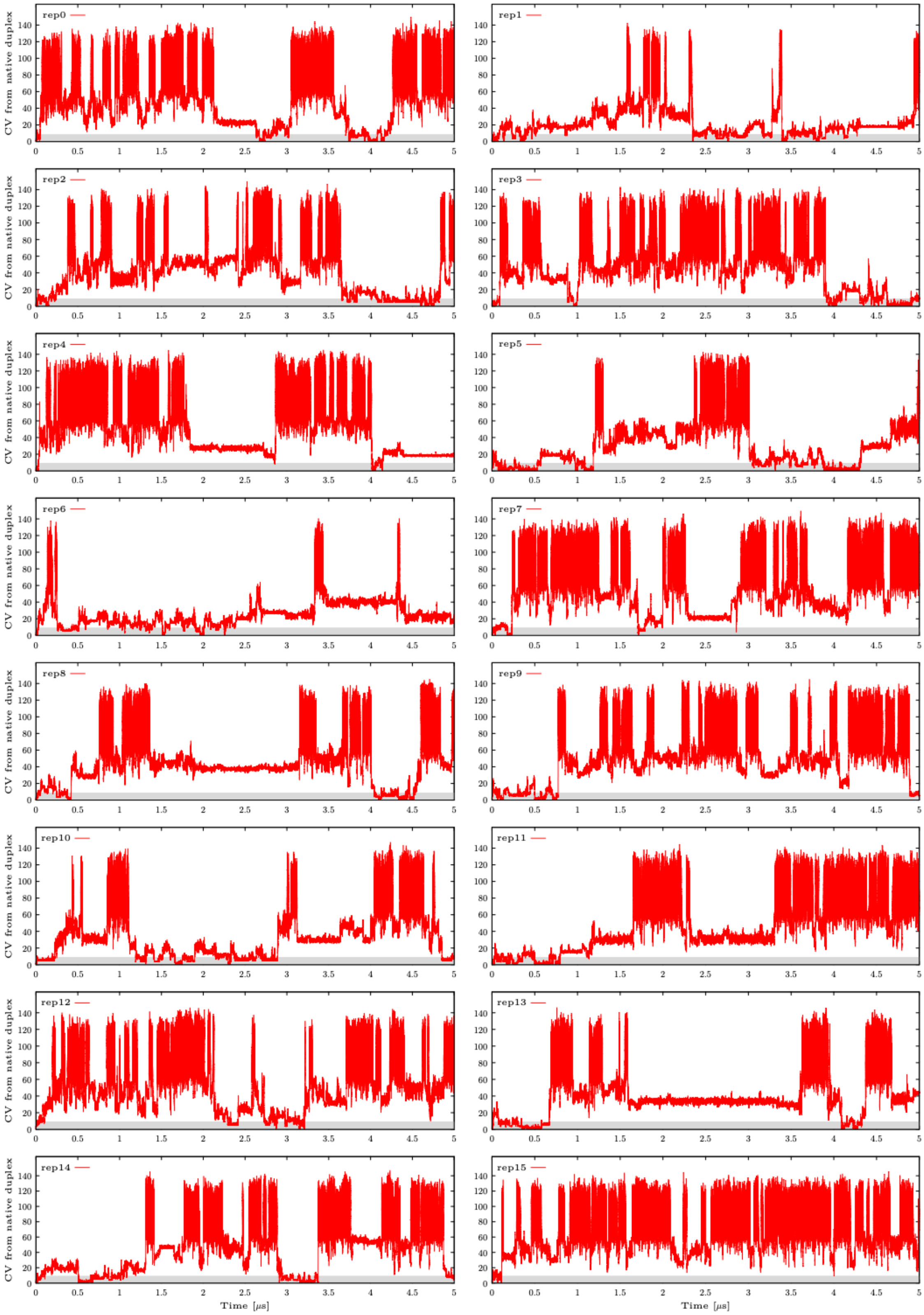

Figure continuing on the next page

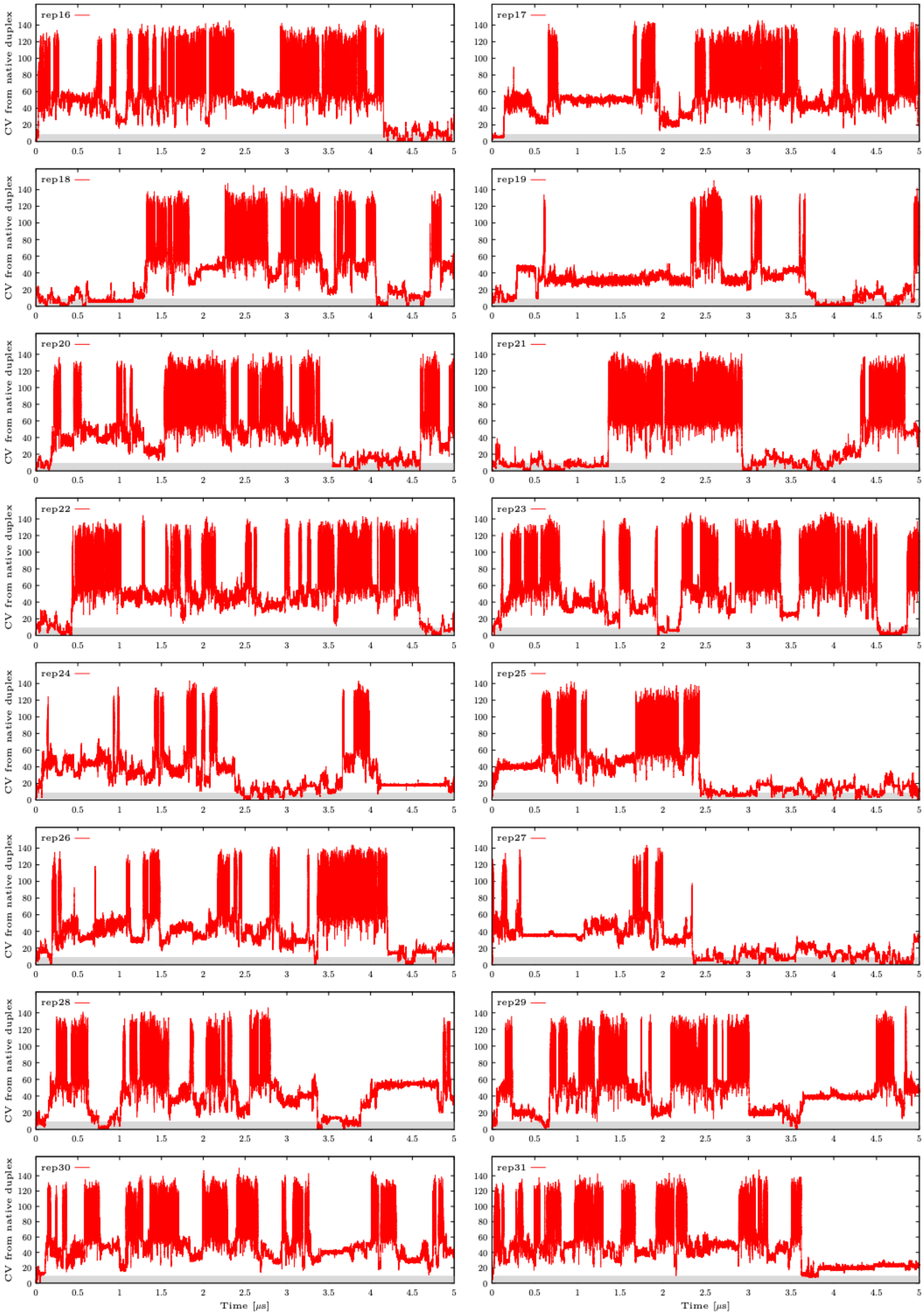

Figure S16. Calculated CV relative to the native state for each of the 32 continuous (demultiplexed) replicas from the 5 μs PT-OPES simulation of the short r(GGCC)$_2$ duplex with the OL3$_{CP}$–NBfix19 FF. See Figure S15 for definitions and analysis details.

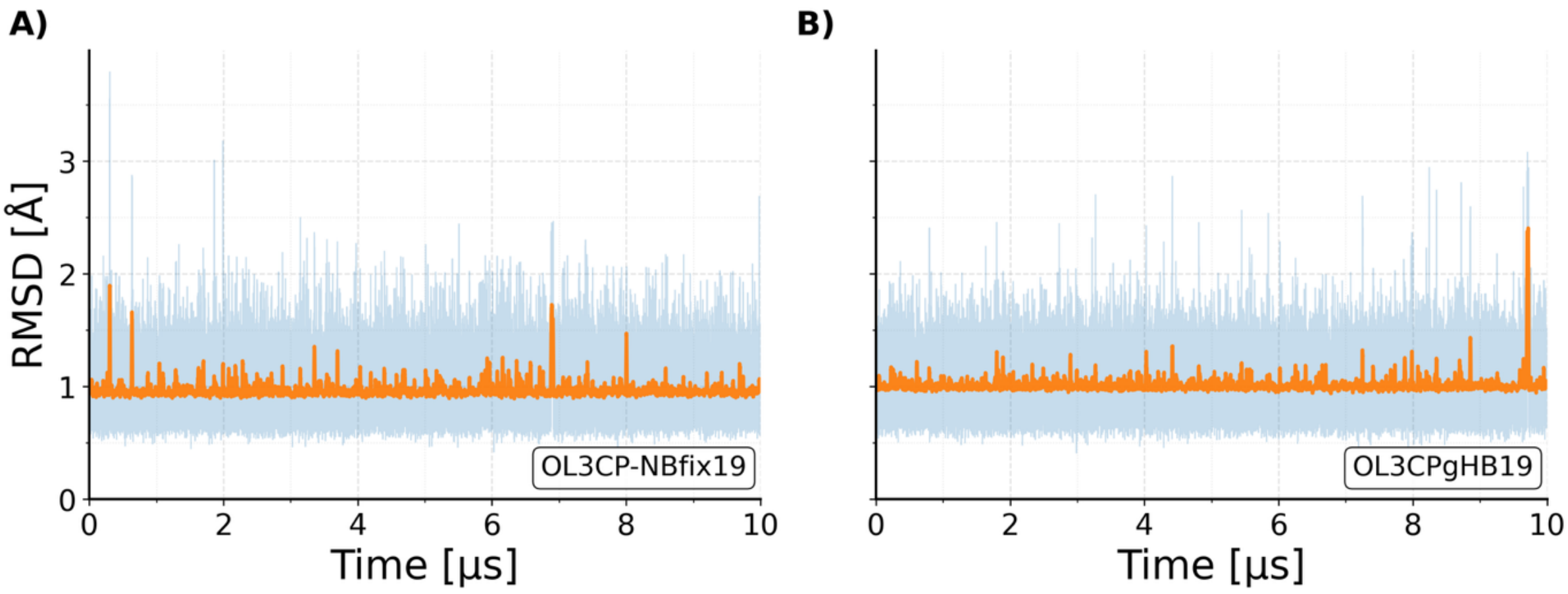

Figure S17. Evolution of the RMSD from two 10 µs standard MD simulations of the GAGA TL using (A) $OL3_{CP}$–NBfix19 and (B) $OL3_{CP}$–gHBfix19 FFs. Light blue traces show the actual RMSD fluctuations, whereas orange traces show the centered running average (10 ns windows).

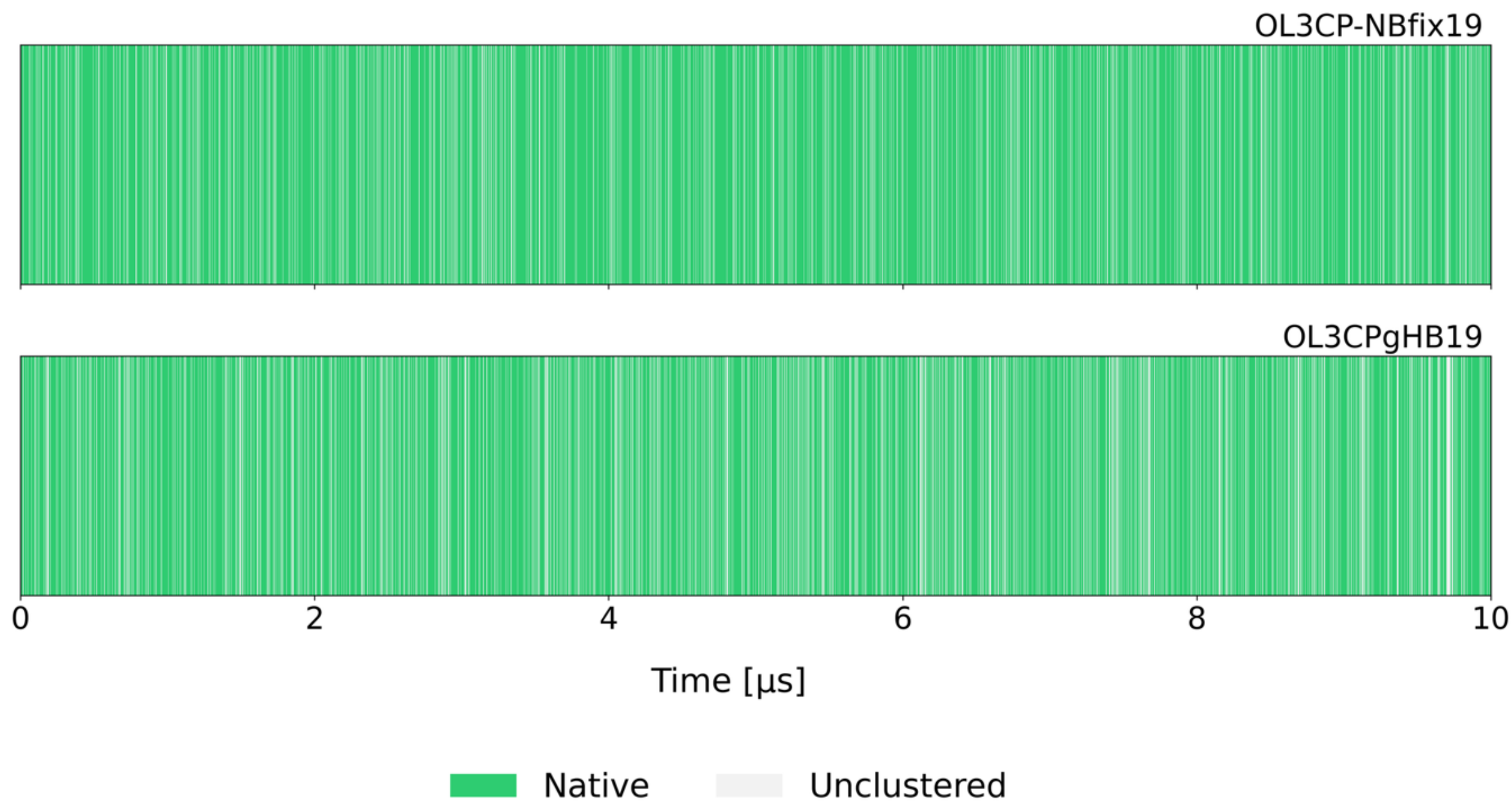

Figure S18. Time evolution of structural states sampled during two standard MD simulations of the GAGA TL using (A) $OL3_{CP}$–NBfix19 and (B) $OL3_{CP}$–gHBfix19 FFs. Each frame is classified as either native (green; based on the presence of all signature loop interactions, see, e.g., Ref. [23] for details) or unclustered (grey; representing conformations that do not satisfy the native-state criteria).

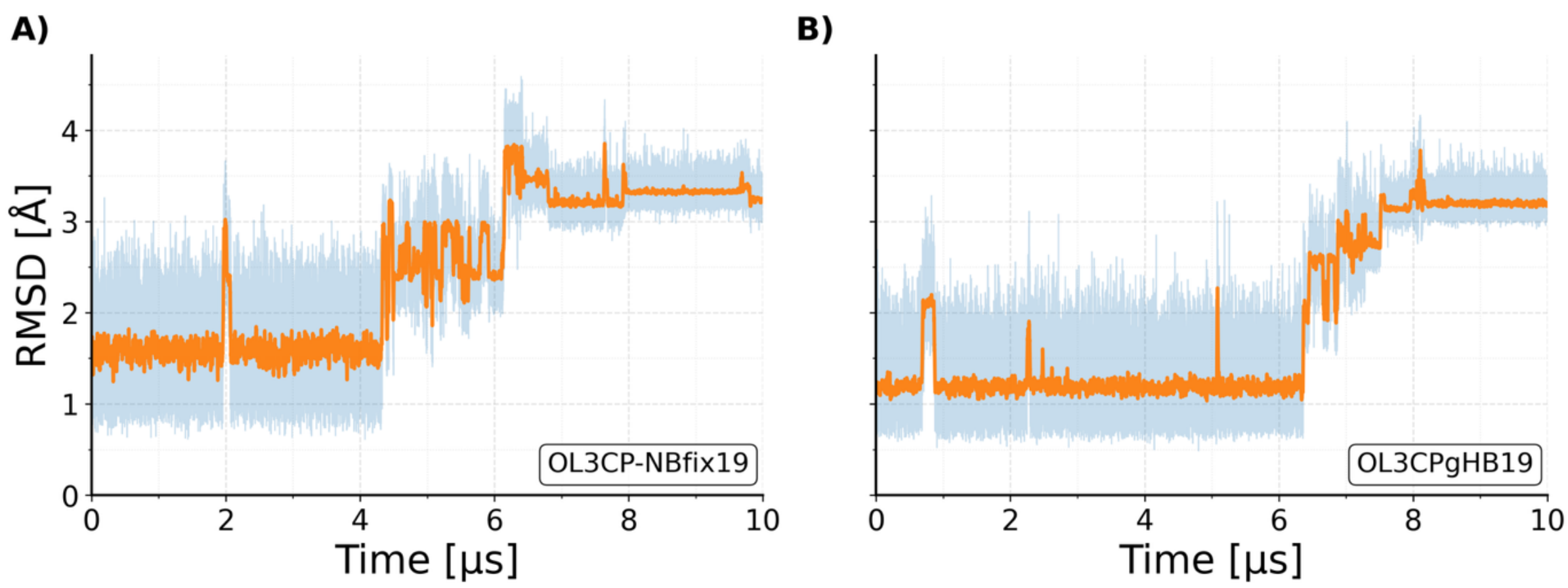

Figure S19. Evolution of the RMSD from two 10 μs standard MD simulations of the 14-mer UUCG TL (PDB ID 2KOC[8]) using (A) $OL3_{CP}$–NBfix19 and (B) $OL3_{CP}$–gHBfix19 FFs. See Figure S17 for more details.

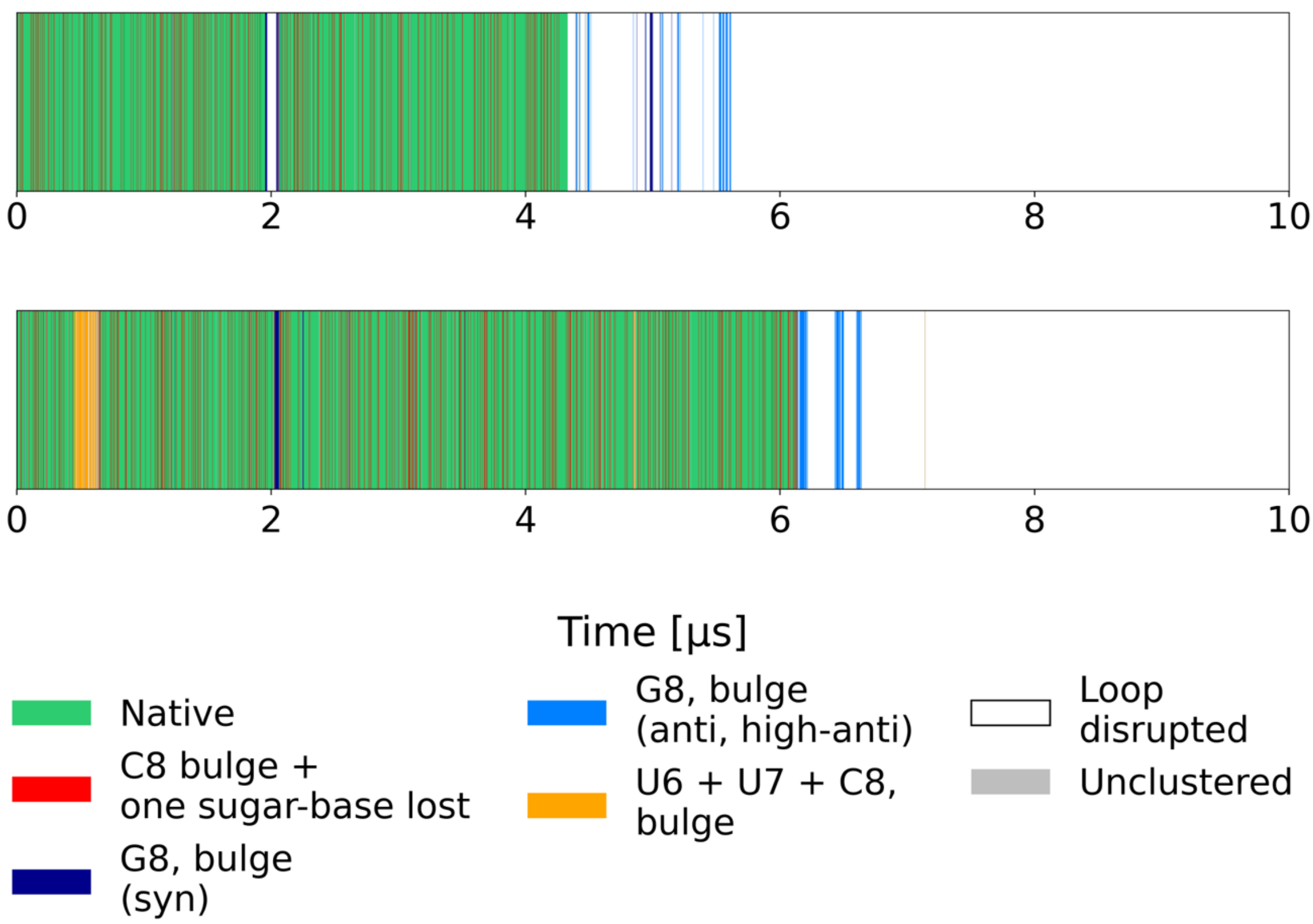

Figure S20. Time evolution of key structural states sampled during two standard MD simulations of 14-mer UUCG TL (PDB ID 2KOC[8]) using (A) $OL3_{CP}$–NBfix19 and (B) $OL3_{CP}$–gHBfix19 FFs. See Ref.[18] for details about the structural states.

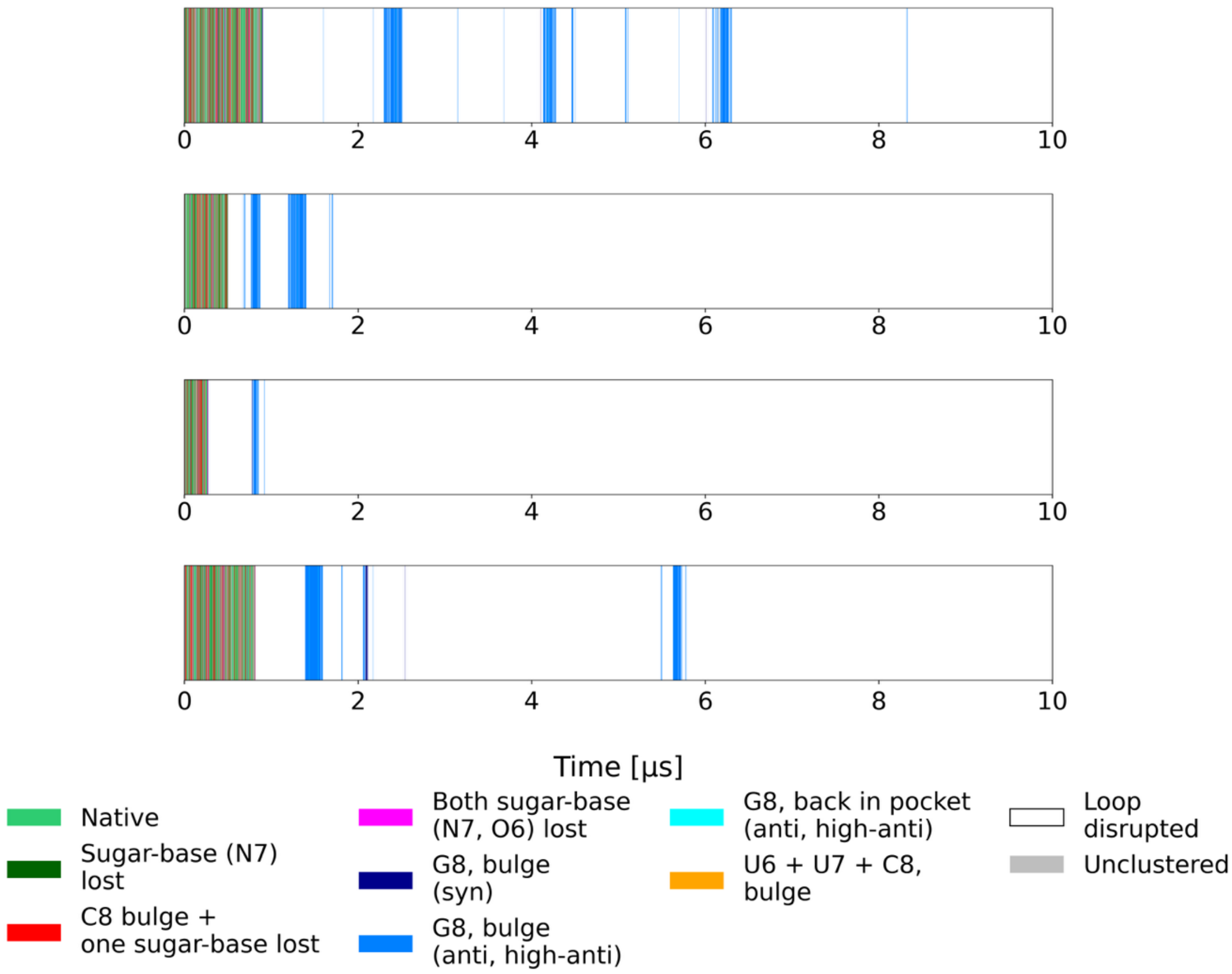

Figure S21. Time evolution of key structural states sampled during four independent standard MD simulations of 8-mer UUCG TL using OL3-vdW7-PAK FF. See Ref.[18] for details about the structural states.

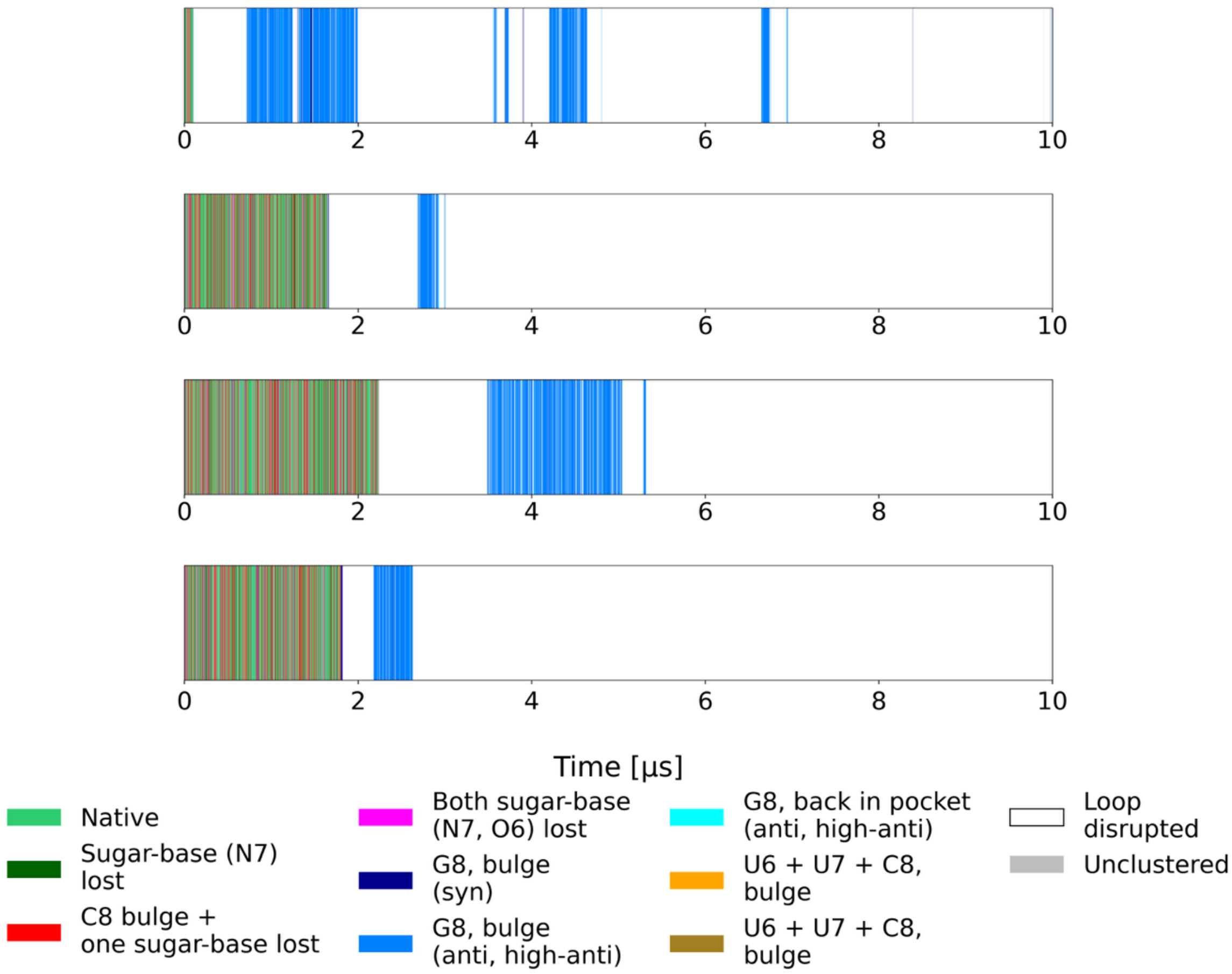

Figure S22. Time evolution of key structural states sampled during four independent standard MD simulations of 10-mer UUCG TL using OL3-vdW7-PAK FF. See Ref.[18] for details about the structural states.

REFERENCES

(1) Cornell, W. D.; Cieplak, P.; Bayly, C. I.; Gould, I. R.; Merz, K. M.; Ferguson, D. M.; Spellmeyer, D. C.; Fox, T.; Caldwell, J. W.; Kollman, P. A. A second generation force field for the simulation of proteins, nucleic acids, and organic molecules (vol 117, pg 5179, 1995). *J Am Chem Soc* **1996**, *118* (9), 2309-2309.
(2) Wang, J. M.; Cieplak, P.; Kollman, P. A. How well does a restrained electrostatic potential (RESP) model perform in calculating conformational energies of organic and biological molecules? *J Comp Chem* **2000**, *21* (12), 1049-1074.
(3) Perez, A.; Marchan, I.; Svozil, D.; Sponer, J.; Cheatham, T. E.; Laughton, C. A.; Orozco, M. Refinement of the AMBER Force Field for Nucleic Acids: Improving the Description of Alpha/Gamma Conformers. *Biophys J* **2007**, *92* (11), 3817-3829, Article. DOI: 10.1529/biophysj.106.097782.
(4) Zgarbova, M.; Otyepka, M.; Sponer, J.; Mladek, A.; Banas, P.; Cheatham, T. E.; Jurecka, P. Refinement of the Cornell et al. Nucleic Acids Force Field Based on Reference Quantum Chemical Calculations of Glycosidic Torsion Profiles. *J Chem Theory Comput* **2011**, *7* (9), 2886-2902. DOI: 10.1021/ct200162x.
(5) Steinbrecher, T.; Latzer, J.; Case, D. A. Revised AMBER Parameters for Bioorganic Phosphates. *J Chem Theory Comput* **2012**, *8* (11), 4405-4412.
(6) Mlynsky, V.; Kuhrova, P.; Zgarbova, M.; Jurecka, P.; Walter, N. G.; Otyepka, M.; Sponer, J.; Banas, P. Reactive conformation of the active site in the hairpin ribozyme achieved by molecular dynamics simulations with epsilon/zeta force field reparametrizations. *J Phys Chem B* **2015**, *119* (11), 4220-4229. DOI: 10.1021/jp512069n.
(7) Kim, H.; Pak, Y. Ensemble-Based Precision Refinement of All-Atom Nucleic Acid Force Fields Guided by NMR NOE Pair-Distance Measurements. *J Chem Theory Comput* **2025**, *21* (21), 11245-11258. DOI: 10.1021/acs.jctc.5c01075.
(8) Nozinovic, S.; Furtig, B.; Jonker, H. R.; Richter, C.; Schwalbe, H. High-resolution NMR structure of an RNA model system: the 14-mer cUUCGg tetraloop hairpin RNA. *Nucleic Acids Res* **2010**, *38* (2), 683-694. DOI: 10.1093/nar/gkp956.
(9) Invernizzi, M.; Parrinello, M. Rethinking Metadynamics: From Bias Potentials to Probability Distributions. *J Phys Chem Lett* **2020**, *11* (7), 2731-2736. DOI: 10.1021/acs.jpclett.0c00497.
(10) Invernizzi, M.; Parrinello, M. Exploration vs Convergence Speed in Adaptive-Bias Enhanced Sampling. *J Chem Theory Comput* **2022**, *18* (6), 3988-3996. DOI: 10.1021/acs.jctc.2c00152.
(11) Kuhrova, P.; Mlynsky, V.; Zgarbova, M.; Krepl, M.; Bussi, G.; Best, R. B.; Otyepka, M.; Sponer, J.; Banas, P. Improving the Performance of the Amber RNA Force Field by Tuning the Hydrogen-Bonding Interactions. *J Chem Theory Comput* **2019**, *15* (5), 3288-3305. DOI: 10.1021/acs.jctc.8b00955.
(12) Abraham, M. J.; Murtola, T.; Schulz, R.; Páll, S.; Smith, J. C.; Hess, B.; Lindahl, E. GROMACS: High performance molecular simulations through multi-level parallelism from laptops to supercomputers. *SoftwareX* **2015**, *1*, 19-25.
(13) Izadi, S.; Onufriev, A. V. Accuracy limit of rigid 3-point water models. *J Chem Phys* **2016**, *145* (7). DOI: Artn 074501
10.1063/1.4960175.

(14) Sengupta, A.; Li, Z.; Song, L. F.; Li, P.; Merz, K. M., Jr. Parameterization of Monovalent Ions for the OPC3, OPC, TIP3P-FB, and TIP4P-FB Water Models. *J Chem Inf Model* **2021**, *61* (2), 869-880. DOI: 10.1021/acs.jcim.0c01390 From NLM Medline.
(15) Hess, B.; Bekker, H.; Berendsen, H. J. C.; Fraaije, J. G. E. M. LINCS: A Linear Constraint Solver for Molecular Simulations. *J Comp Chem* **1997**, *18* (12), 1463-1472. DOI: 10.1002/(sici)1096-987x(199709)18:12<1463::aid-jcc4>3.0.co;2-h.
(16) Bussi, G.; Donadio, D.; Parrinello, M. Canonical Sampling through Velocity Rescaling. *J Chem Phys* **2007**, *126* (1), 014101. DOI: doi:http://dx.doi.org/10.1063/1.2408420.
(17) Mrazikova, K.; Mlynsky, V.; Kuhrova, P.; Pokorna, P.; Kruse, H.; Krepl, M.; Otyepka, M.; Banas, P.; Sponer, J. UUCG RNA Tetraloop as a Formidable Force-Field Challenge for MD Simulations. *J Chem Theory Comput* **2020**, *16* (12), 7601-7617. DOI: 10.1021/acs.jctc.0c00801.
(18) Mlynsky, V.; Kührová, P.; Pykal, M.; Krepl, M.; Stadlbauer, P.; Otyepka, M.; Banás, P.; Sponer, J. Can We Ever Develop an Ideal RNA Force Field? Lessons Learned from Simulations of the UUCG RNA Tetraloop and Other Systems. *J Chem Theory Comput* **2025**, *21* (8), 4183-4202. DOI: 10.1021/acs.jctc.4c01357.
(19) Mlynsky, V.; Kuhrova, P.; Kuhr, T.; Otyepka, M.; Bussi, G.; Banas, P.; Sponer, J. Fine-Tuning of the AMBER RNA Force Field with a New Term Adjusting Interactions of Terminal Nucleotides. *J Chem Theory Comput* **2020**, *16* (6), 3936-3946. DOI: 10.1021/acs.jctc.0c00228.
(20) Klosterman, P. S.; Shah, S. A.; Steitz, T. A. Crystal structures of two plasmid copy control related RNA duplexes: An 18 base pair duplex at 1.20 A resolution and a 19 base pair duplex at 1.55 A resolution. *Biochemistry* **1999**, *38* (45), 14784-14792.
(21) Dockbregeon, A. C.; Chevrier, B.; Podjarny, A.; Johnson, J.; Debear, J. S.; Gough, G. R.; Gilham, P. T.; Moras, D. Crystallographic Structure of an Rna Helix - [U(Ua)6a]2. *J Mol Biol* **1989**, *209* (3), 459-474.
(22) Bottaro, S.; Di Palma, F.; Bussi, G. The role of nucleobase interactions in RNA structure and dynamics. *Nucleic Acids Res* **2014**, *42* (21), 13306-13314. DOI: 10.1093/nar/gku972.
(23) Kuhrova, P.; Mlynsky, V.; Frebort, I.; Frebortova, J.; Otyepka, M.; Sponer, J.; Banas, P. Toward Accurate RNA Folding Thermodynamics: Evaluation of Enhanced Sampling Methods for Force Field Benchmarking. *J Chem Theory Comput* **2026**, *22* (10), 5247-5258. DOI: 10.1021/acs.jctc.6c00108.
